# N4-acetylcytidine modification bridges metabolic reprogramming and immune evasion in cancer: mechanisms and therapeutic implications

**DOI:** 10.3389/fimmu.2026.1820962

**Published:** 2026-06-29

**Authors:** Ming-Zhu Jin, Wen Di

**Affiliations:** 1 Department of Obstetrics and Gynecology, Renji Hospital, School of Medicine, Shanghai Jiao Tong University, Shanghai, China; 2 Shanghai Key Laboratory of Gynecologic Oncology, Renji Hospital, School of Medicine, Shanghai Jiao Tong University, Shanghai, China

**Keywords:** N4-acetylcytidine (AC4C), glycolysis, immune evasion, metabolic reprogramming, immunotherapy, N-acetyltransferase 10 (NAT10), tumor microenvironment

## Abstract

N4-acetylcytidine (ac4C) is an evolutionarily conserved RNA modification catalyzed by N-acetyltransferase 10 (NAT10), representing the sole known acetylation modification in eukaryotic mRNA. Recent studies have revealed that ac4C modification plays multifaceted roles in cancer progression by regulating mRNA stability and translation efficiency. Notably, emerging evidence demonstrates that NAT10-mediated ac4C modification simultaneously orchestrates tumor metabolic reprogramming and immune evasion, two hallmarks of cancer that are increasingly recognized as interconnected processes. In metabolic regulation, ac4C modification enhances the stability and translation of key glycolytic enzymes, including hexokinase (HK1/2), enolase 1 (ENO1), lactate dehydrogenase A (LDHA), and phosphoglycerate mutase 1 (PGAM1), thereby promoting the Warburg effect. Concurrently, ac4C modification facilitates immune evasion through multiple mechanisms, including upregulation of PD-L1 expression, suppression of T cell function, and inhibition of type I interferon signaling. Importantly, glycolysis-driven lactate accumulation creates an immunosuppressive tumor microenvironment, suggesting that ac4C serves as a molecular bridge connecting metabolic reprogramming to immune escape. Targeting NAT10 with inhibitors such as Remodelin has shown promising preclinical efficacy, particularly when combined with immune checkpoint inhibitors. This review comprehensively summarizes the current understanding of ac4C modification in tumor metabolism and immunity, highlights the metabolic-immune crosstalk mediated by ac4C, and discusses the therapeutic potential of targeting this modification for cancer treatment. We also highlight emerging controversies regarding ac4C stoichiometry in human mRNA, cell-type-specific functions of ac4C in the tumor microenvironment, and the expanding regulatory network encompassing non-coding RNAs and crosstalk with other RNA modifications including m5C, pseudouridine, and m6Am.

## 1 Introduction

The epitranscriptome, comprising over 170 distinct chemical modifications of RNA, has emerged as a critical regulatory layer governing gene expression in both physiological and pathological contexts (1–6). Among these modifications, N6-methyladenosine (m6A) has been most extensively characterized, yet the broader landscape of mRNA modifications, including pseudouridine (Ψ), 5-methylcytidine (m5C), and N4-acetylcytidine (ac4C), is now recognized as equally important in shaping cellular identity and disease states (7–9). In cancer, dysregulation of the epitranscriptome contributes to virtually every hallmark of malignancy, from uncontrolled proliferation to immune evasion, positioning RNA modification enzymes as a new class of oncogenic drivers and therapeutic targets (1–6).

ac4C was initially identified as a conserved modification in transfer RNA (tRNA) and ribosomal RNA (rRNA), where it contributes to translational fidelity and ribosome biogenesis. The landmark study by Arango et al. (10) extended this paradigm to mRNA, demonstrating that ac4C modification of cytidine residues in coding sequences enhances ribosome occupancy and translational efficiency. This discovery established NAT10 as the sole known mRNA acetyltransferase in eukaryotes and opened a new chapter in epitranscriptomic research (11–16). Subsequent work has shown that ac4C modification is not uniformly distributed across the transcriptome but is enriched at specific sequence contexts, suggesting a targeted regulatory mechanism with broad implications for gene expression control.

Cancer cells exhibit profound metabolic alterations to support their rapid proliferation and survival. The Warburg effect, characterized by preferential utilization of aerobic glycolysis even in the presence of oxygen, is one of the most prominent metabolic alterations in cancer and generates the acidic, nutrient-depleted tumor microenvironment that simultaneously fuels tumor growth and suppresses anti-tumor immunity (17–27). Immune evasion, another hallmark of cancer, is achieved through multiple mechanisms including upregulation of immune checkpoint molecules, suppression of cytotoxic lymphocyte function, and remodeling of the tumor microenvironment into an immunosuppressive niche (28–31). Critically, metabolic reprogramming and immune evasion are not independent processes but are deeply intertwined: glycolysis-derived lactate directly suppresses T cell and NK cell function, while hypoxia-driven transcription factors simultaneously activate glycolytic gene programs and immune checkpoint expression.

Recent studies have revealed that NAT10-mediated ac4C modification plays crucial roles in both metabolic reprogramming and immune evasion, positioning it as a molecular bridge between these two cancer hallmarks. In the context of metabolism, ac4C modification has been shown to enhance the stability and translation of key glycolytic enzymes including hexokinase 2 (HK2), enolase 1 (ENO1), lactate dehydrogenase A (LDHA), and phosphoglycerate mutase 1 (PGAM1), thereby driving the Warburg effect across multiple cancer types. In the immune context, NAT10-mediated ac4C modification promotes immune evasion by upregulating PD-L1 expression, suppressing type I interferon signaling, and remodeling the tumor microenvironment to exclude cytotoxic T cells. The convergence of these functions on a single enzymatic axis, NAT10 makes it an exceptionally attractive therapeutic target.

**Figure 1:**
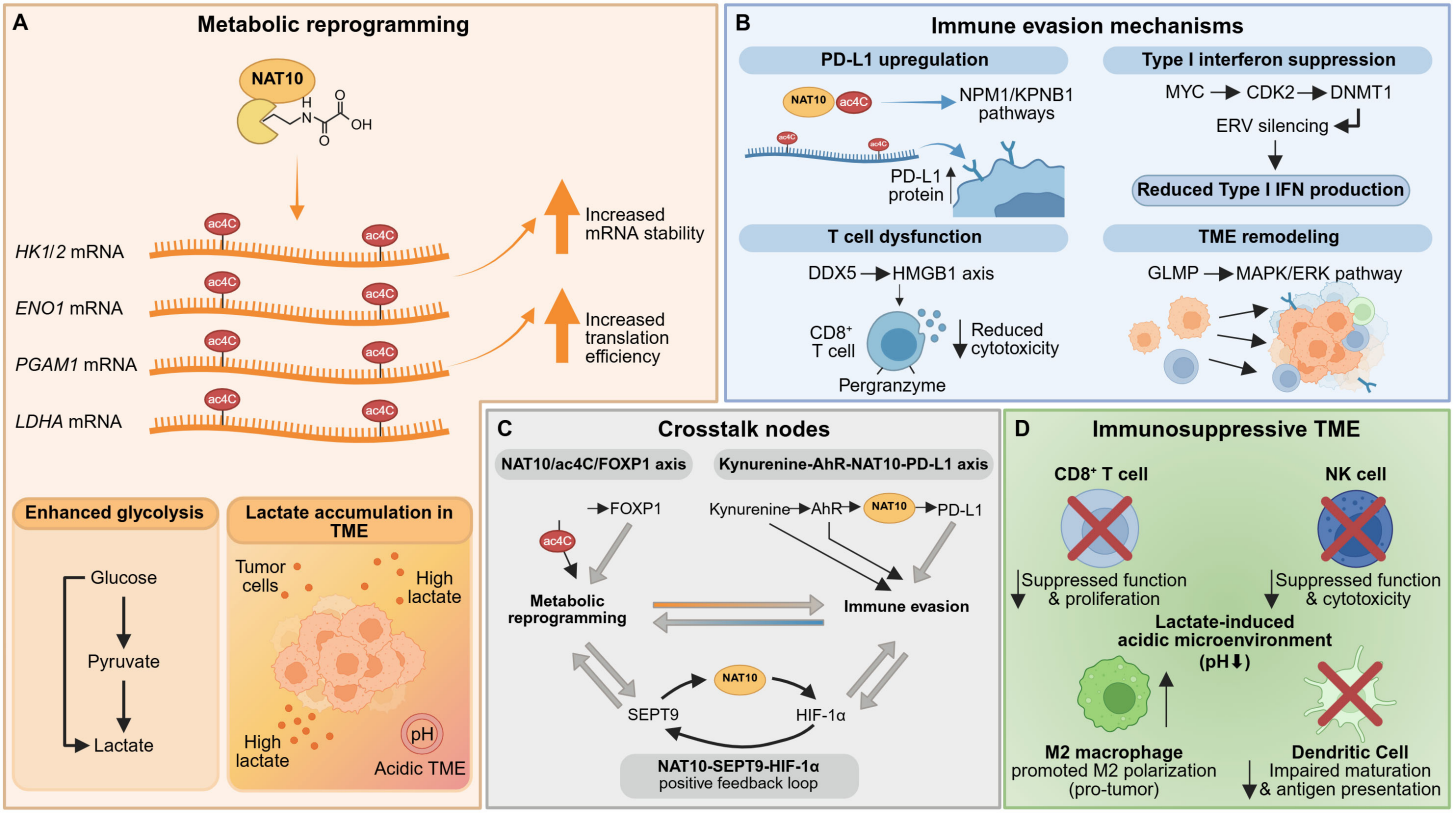
Integrated model of ac4C-mediated metabolic-immune crosstalk in cancer. NAT10-mediated ac4C modification serves as a molecular bridge connecting tumor metabolic reprogramming to immune evasion. **(A)** Metabolic reprogramming: NAT10 catalyzes ac4C modification on mRNAs of key glycolytic enzymes (HK1/2, ENO1, PGAM1, LDHA), enhancing their stability and translation efficiency. This promotes the Warburg effect and lactate accumulation in the tumor microenvironment. **(B)** Immune evasion: ac4C modification promotes immune escape through multiple mechanisms including PD-L1 upregulation (via direct mRNA modification and NPM1/KPNB1 pathways), type I interferon suppression (via MYC/CDK2/DNMT1-mediated ERV silencing), T cell dysfunction (via DDX5/HMGB1 axis), and TME remodeling (via GLMP/MAPK/ERK pathway). **(C)** Crosstalk nodes: Three key molecular nodes connect metabolism to immunity, the NAT10/ac4C/FOXP1 axis (promotes both glycolysis and immunosuppression), the kynurenine-AhR-NAT10-PD-L1 axis (links tryptophan metabolism to immune checkpoint regulation), and the NAT10/SEPT9/HIF-1α positive feedback loop (sustains both glycolytic gene expression and immunosuppressive signaling). **(D)** Immunosuppressive TME: Lactate accumulation creates an acidic microenvironment that directly inhibits CD8+ T cell and NK cell cytotoxicity, promotes M2 macrophage polarization, and impairs dendritic cell maturation. Created with biorender.

Despite the growing body of evidence linking ac4C to cancer progression, a comprehensive review that integrates the metabolic and immunological aspects of ac4C modification is lacking. Existing reviews have focused either on the biochemical mechanisms of ac4C modification or on individual cancer types, without addressing the broader question of how ac4C coordinates metabolic and immune programs to drive malignancy. This review aims to fill this gap by providing a systematic analysis of ac4C modification in tumor glycolytic reprogramming and immune evasion, proposing an integrated model of metabolic-immune crosstalk mediated by ac4C, and discussing the therapeutic implications of targeting this axis. By synthesizing current knowledge across multiple cancer types and biological contexts, we hope to provide a conceptual framework that will guide future research and accelerate the clinical translation of NAT10-targeted therapies (**Figure 1**).

## 2 Molecular mechanisms of ac4C modification

### 2.1 NAT10: the writer enzyme of ac4C

**Figure 2:**
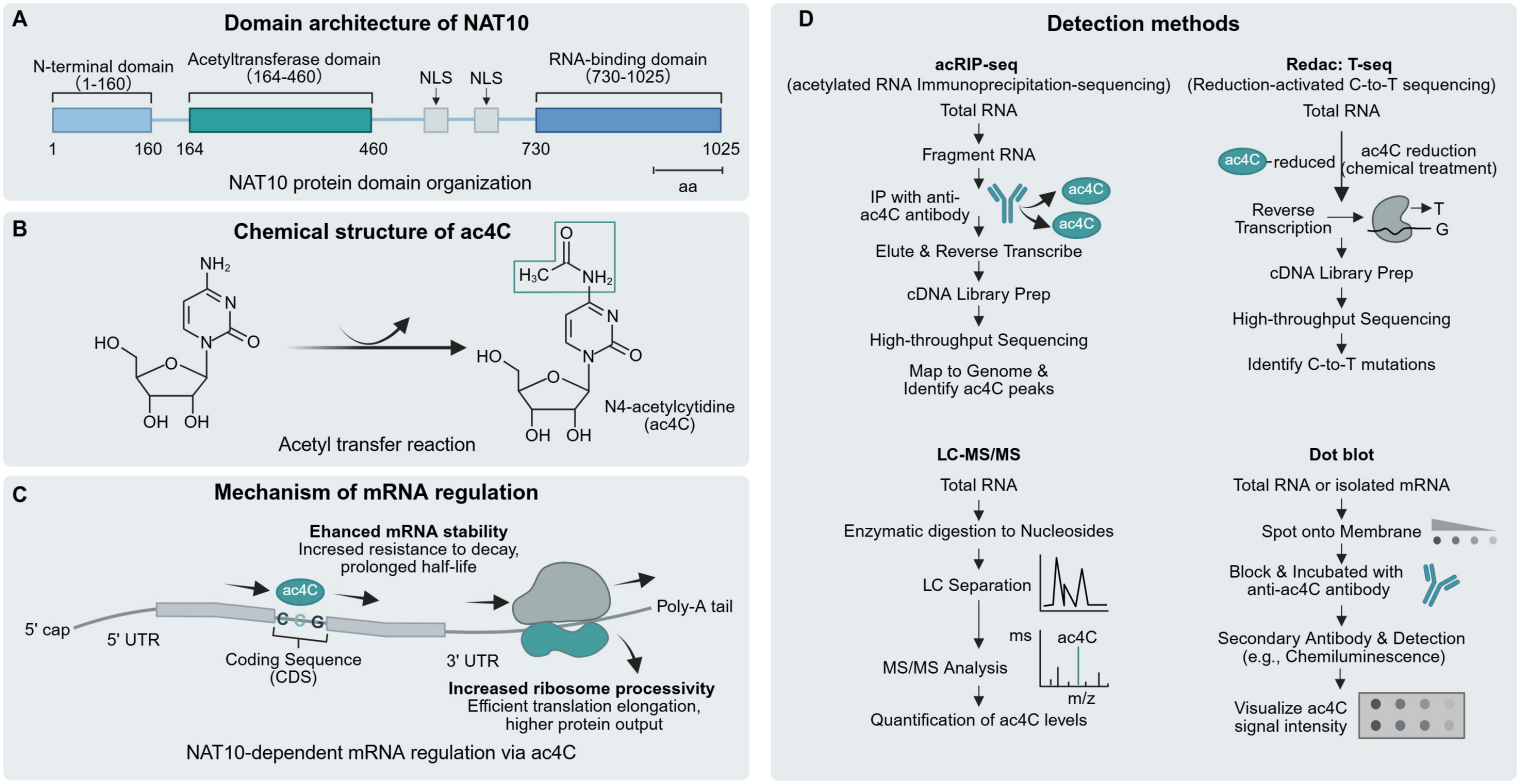
Structural organization of NAT10 and mechanism of ac4C modification. **(A)** Domain structure of human NAT10 protein. NAT10 is a 1,025-amino acid protein containing: N-terminal domain (NTD, aa 1-160) involved in protein-protein interactions; acetyltransferase domain (AT, aa 164-460) responsible for catalyzing acetyl group transfer from acetyl-CoA to cytidine; and C-terminal RNA-binding domain (RBD, aa 730-1025) that recognizes target RNAs. Nuclear localization signals (NLS) are indicated. **(B)** Chemical structure of ac4C modification. N4-acetylcytidine (ac4C) is formed by the addition of an acetyl group (-COCH3) to the N4 position of cytidine. The modification is catalyzed by NAT10 using acetyl-CoA as the acetyl donor. **(C)** Mechanism of ac4C-mediated mRNA regulation. ac4C modification within the coding sequence (CDS) of mRNA enhances: (1) mRNA stability by protecting against degradation; (2) translation efficiency by promoting ribosome processivity. The consensus motif for ac4C modification is CCG. **(D)** Detection methods for ac4C modification. Current approaches include: acRIP-seq (antibody-based immunoprecipitation), RedaC:T-seq (chemical reduction-based sequencing for single-nucleotide resolution), LC-MS/MS (quantitative mass spectrometry), and dot blot assays (semi-quantitative screening). Created with biorender.

N-acetyltransferase 10 (NAT10) is a highly conserved protein that serves as the sole identified writer enzyme for ac4C modification in eukaryotes (32–38). Human NAT10 is a 1,025-amino acid protein localized predominantly in the nucleolus, with a domain architecture that reflects its multifunctional nature: an N-terminal acetyltransferase domain harboring the catalytic GNAT fold, a central HEAT repeat domain mediating protein-protein interactions, and a C-terminal RNA-binding domain that confers substrate specificity. Structural studies have revealed that the acetyltransferase domain coordinates acetyl-CoA binding and positions the cytidine substrate for nucleophilic attack, while the HEAT repeats facilitate interaction with the adapter protein THUMPD1 (Tan1 in yeast), which is required for tRNA but not mRNA modification. NAT10 is predominantly expressed in proliferating cells and is frequently overexpressed in cancer, where its elevated activity drives both transcriptional and post-transcriptional gene regulatory programs (**Figure 2**).

NAT10 exhibits dual enzymatic activities, functioning as both an RNA acetyltransferase and a protein acetyltransferase, a duality that expands its regulatory reach well beyond RNA modification (32–38). As an RNA acetyltransferase, NAT10 catalyzes ac4C modification on tRNA, rRNA, and mRNA, with each substrate class requiring distinct cofactors and sequence contexts. As a protein acetyltransferase, NAT10 modifies histones and non-histone proteins including p53, SIRT1, and nucleophosmin 1 (NPM1), thereby influencing chromatin organization, DNA damage responses, and nucleolar function. This dual activity means that pharmacological inhibition of NAT10, for example with the small molecule Remodelin, simultaneously disrupts RNA modification and protein acetylation programs, potentially amplifying therapeutic effects while also complicating the interpretation of mechanism-of-action studies.

The mechanism by which NAT10 recognizes and modifies specific cytidine residues in mRNA remains an active area of investigation (37, 39–42). Unlike tRNA and rRNA modification, which requires the adapter protein THUMPD1 to position the substrate, mRNA modification by NAT10 appears to rely on sequence context and RNA secondary structure. Computational prediction tools including PACES, ERNIE-ac4C, and Caps-ac4C have identified consensus sequence motifs enriched at ac4C sites, suggesting that NAT10 recognizes specific RNA features rather than modifying cytidines indiscriminately. Emerging evidence also suggests that NAT10 may be recruited to specific mRNA targets through interactions with RNA-binding proteins or through association with actively translating ribosomes, though the precise determinants of substrate selectivity remain to be fully elucidated. Computational analyses of transcriptome-wide ac4C mapping data have identified a CCG trinucleotide as the consensus sequence motif preferentially modified by NAT10, although the precise structural basis for this sequence preference remains to be fully elucidated (43, 44).

### 2.2 Readers and erasers: current knowledge and gaps

In contrast to the well-characterized m6A modification system, which includes multiple writer, reader, and eraser proteins, the ac4C regulatory machinery remains incompletely understood. To date, no dedicated ac4C reader protein has been definitively identified, that is, no protein has been shown to specifically recognize and bind ac4C-modified RNA to transduce a downstream signal in the manner that YTHDF proteins read m6A. The functional consequences of ac4C modification, enhanced translational efficiency and mRNA stability, may therefore arise from direct effects on ribosome-mRNA interactions rather than from reader-mediated signaling, though this distinction remains to be rigorously tested. The absence of known readers represents a significant gap in our understanding of ac4C biology and limits the development of targeted interventions that could modulate ac4C signaling without affecting NAT10’s enzymatic activity.

Similarly, no eraser enzyme capable of removing ac4C modification has been conclusively identified, raising the fundamental question of whether ac4C is a reversible or irreversible mark. If ac4C is irreversible, its regulation would occur primarily at the level of NAT10 expression and activity, and the modification would persist until the modified RNA is degraded. Alternatively, ac4C may be dynamically regulated by as-yet-unidentified deacetylases, analogous to the FTO and ALKBH5 erasers of m6A. Resolving this question has important therapeutic implications: if ac4C is reversible, eraser enzymes could serve as alternative drug targets, while if it is irreversible, therapeutic strategies must focus on inhibiting NAT10 or blocking the downstream consequences of ac4C modification. To overcome these gaps, several experimental strategies have been proposed for the systematic identification of ac4C readers and erasers. For reader identification, RNA pull-down assays using synthetic ac4C-modified RNA oligonucleotides as bait, coupled with quantitative mass spectrometry-based proteomics, represent a powerful unbiased approach to capture proteins that selectively bind ac4C-containing sequences. Complementary genetic screens, such as CRISPR-Cas9 loss-of-function screens in cell lines where ac4C-dependent phenotypes (e.g., mRNA stability, translational efficiency, or drug sensitivity) can be quantitatively measured, could identify candidate reader proteins whose depletion phenocopies ac4C loss. For eraser identification, enzymatic activity assays using ac4C-modified RNA substrates, combined with fractionation of nuclear and cytoplasmic extracts, may reveal deacetylase activities that have not yet been linked to ac4C. These approaches, analogous to those that successfully identified m6A readers (e.g., YTHDF proteins) and erasers (FTO, ALKBH5), provide a clear roadmap for completing the ac4C regulatory machinery (44, 45).

### 2.3 Detection methods for ac4C modification

The development of sensitive and specific methods for detecting ac4C modification has been instrumental in advancing the field, and several complementary approaches are currently employed, each with distinct strengths and limitations.

Antibody-based immunoprecipitation sequencing (acRIP-seq) was the first transcriptome-wide method for mapping ac4C and remains widely used. In this approach, anti-ac4C antibodies enrich ac4C-containing RNA fragments, which are then subjected to high-throughput sequencing to identify modification sites at the transcript level. While acRIP-seq provides a global view of the ac4C landscape and is compatible with standard RNA-seq workflows, its resolution is limited to approximately 100–200 nucleotides, and the specificity of available antibodies has been questioned in some contexts.

Chemical-based sequencing methods offer improved resolution by exploiting the unique reactivity of ac4C. The reduction-based ac4C sequencing method (RedaC:T-seq) treats RNA with sodium cyanoborohydride (NaBH3CN), which selectively reduces ac4C to tetrahydro-ac4C, causing reverse transcriptase to misread the modified nucleotide and introduce a C-to-T transition at ac4C sites. This approach enables single-nucleotide resolution mapping of ac4C across the transcriptome and has been validated in multiple organisms, though it requires careful optimization to minimize background signal from non-specific reductions.

Mass spectrometry, particularly liquid chromatography-tandem mass spectrometry (LC-MS/MS), provides quantitative measurement of global ac4C levels in RNA samples (46) and is especially valuable for comparing ac4C abundance across experimental conditions or cancer types. While this method cannot identify specific modification sites, it offers unparalleled sensitivity and accuracy for bulk quantification and is often used to validate findings from sequencing-based approaches.

For rapid, semi-quantitative assessment of ac4C levels, dot blot assays using anti-ac4C antibodies provide a practical screening tool that is often employed for initial validation before committing to more resource-intensive sequencing experiments. Computational prediction tools including PACES, ERNIE-ac4C, XG-ac4C, and Caps-ac4C complement experimental methods by enabling genome-wide prediction of ac4C sites based on sequence features, facilitating hypothesis generation and prioritization of candidate modification sites for experimental validation.

Each detection method has its strengths and limitations, and researchers typically employ multiple complementary approaches to comprehensively characterize ac4C modification patterns. The continued development of more sensitive, specific, and high-throughput detection technologies, including single-molecule sequencing approaches that may enable direct detection of ac4C without chemical treatment, will be essential for advancing our understanding of ac4C biology in cancer and other diseases.

Beyond these established methods, several emerging technologies are expanding the ac4C detection toolkit. Single-cell ac4C sequencing (sc-ac4C-seq), adapted from bulk RedaC:T-seq, enables transcriptome-wide ac4C profiling at single-cell resolution, allowing dissection of cell-type-specific modification patterns within heterogeneous tumor tissues. Spatial ac4C sequencing integrates ac4C detection with spatial transcriptomics platforms (e.g., 10x Visium), enabling simultaneous mapping of ac4C modification and spatial gene expression, which is particularly valuable for characterizing ac4C dynamics across distinct tumor microenvironmental niches. Single-molecule real-time sequencing (SMRT-seq) by Pacific Biosciences offers the potential for direct, base-modification-aware sequencing of ac4C without chemical conversion steps, though its sensitivity for ac4C detection in mRNA remains under active development. These next-generation methods promise to resolve the cell-type and spatiotemporal specificity of ac4C modification that bulk sequencing approaches cannot capture. Importantly, the field has recently been confronted with a significant controversy: a 2024 study published in Molecular Cell reported that ac4C modification may be absent or present at extremely low stoichiometry in human mRNA, challenging the foundational findings of Arango et al. (2018). The authors argued that previous acRIP-seq signals may reflect antibody cross-reactivity or technical artifacts rather than bona fide mRNA acetylation. This controversy underscores the critical importance of orthogonal validation using multiple detection methods, and highlights the need for improved antibody specificity and chemical sequencing approaches with rigorous controls. Resolution of this debate is essential for the field, as it directly impacts the interpretation of all downstream functional studies linking ac4C to cancer biology (47–49).

## 3 ac4C modification in tumor glycolytic reprogramming

Metabolic reprogramming is a hallmark of cancer, with the Warburg effect, preferential utilization of glycolysis even under aerobic conditions, being one of the most prominent metabolic alterations in tumor cells (32–38). This metabolic shift is not merely a passive consequence of rapid proliferation but an active program driven by oncogenic signaling that confers multiple advantages: rapid ATP generation, biosynthetic precursor production, and creation of an acidic microenvironment that suppresses immune surveillance. Accumulating evidence now establishes NAT10-mediated ac4C modification as a central driver of this glycolytic program, operating through direct post-transcriptional stabilization of multiple glycolytic enzyme mRNAs and through indirect regulation of the hypoxia-inducible transcriptional network.

### 3.1 ac4C modification of hexokinases (HK1/HK2)

Hexokinases catalyze the first committed step of glycolysis, phosphorylating glucose to glucose-6-phosphate. Among the four hexokinase isoforms, HK1 and HK2 are frequently overexpressed in cancer cells and are associated with poor prognosis across multiple tumor types (50–56). HK2 is considered a cancer-specific isoform because its expression is largely restricted to embryonic tissues and cancer cells in adults, making it an attractive therapeutic target. The mechanisms driving HK2 overexpression in cancer are diverse, encompassing transcriptional activation by HIF-1α and c-Myc, post-translational stabilization, and as recently established post-transcriptional regulation through ac4C modification.

ac4C modification regulates hexokinase expression through a nutrient-sensing mechanism that couples glucose availability to glycolytic enzyme output. When glucose is abundant, NAT10 expression is upregulated, leading to enhanced ac4C modification of HK2 mRNA, increased HK2 protein levels, and accelerated glycolytic flux; conversely, glucose deprivation reduces NAT10 activity and HK2 expression (57). This feed-forward loop positions ac4C modification as a metabolic sensor that amplifies glycolysis precisely when substrate is available, a property that would confer a competitive growth advantage in the nutrient-rich regions of a tumor. The same logic extends to HK1: ac4C modification stabilizes HK1 mRNA and enhances its translation, promoting glycolysis and suppressing apoptosis (58). That both HK1 and HK2, the two cancer-relevant hexokinase isoforms are ac4C targets underscores the breadth of NAT10’s control over the committed entry point of glycolysis, and suggests that ac4C modification may be a general mechanism for sustaining hexokinase expression across diverse tumor contexts.

### 3.2 ac4C modification of enolase 1

Enolase 1 (ENO1) catalyzes the conversion of 2-phosphoglycerate to phosphoenolpyruvate in the penultimate step of glycolysis. ENO1 is frequently overexpressed in various cancers and has been implicated not only in glycolysis but also in plasminogen binding, DNA repair, and stress responses, reflecting its moonlighting functions beyond the glycolytic pathway (26, 59). The regulation of ENO1 by ac4C modification adds another layer to its complex biology in cancer.

ac4C modification of ENO1 mRNA enhances its stability and translational efficiency, increasing ENO1 protein levels and accelerating the conversion of 2-phosphoglycerate to phosphoenolpyruvate (60). The downstream consequences extend beyond glycolytic flux: elevated phosphoenolpyruvate feeds the pentose phosphate pathway and amino acid biosynthesis, supporting the broader anabolic demands of proliferating tumor cells. Critically, this post-transcriptional stabilization also suppresses apoptosis, NAT10 knockdown not only impairs glycolysis but sensitizes tumor cells to apoptotic stimuli, revealing that ac4C modification of ENO1 mRNA simultaneously sustains energy metabolism and cell survival. This dual function makes the NAT10-ENO1 axis a particularly attractive vulnerability in glycolysis-addicted tumors.

### 3.3 ac4C modification of lactate dehydrogenase A

Lactate dehydrogenase A (LDHA) catalyzes the final step of aerobic glycolysis, converting pyruvate to lactate while regenerating NAD+ to sustain glycolytic flux. LDHA is a critical enzyme for maintaining the Warburg effect and is frequently overexpressed in cancer, where elevated LDHA activity correlates with poor prognosis, resistance to therapy, and an immunosuppressive tumor microenvironment (61–63). The regulation of LDHA by ac4C modification is particularly significant because LDHA-derived lactate directly suppresses anti-tumor immunity, creating a direct mechanistic link between ac4C-driven metabolism and immune evasion.

ac4C modification of LDHA mRNA enhances its stability and translation, increasing LDHA protein levels and lactate output, a consequence that is particularly significant because LDHA-derived lactate directly suppresses anti-tumor immunity by impairing T cell receptor signaling, reducing effector cytokine production, and promoting regulatory T cell expansion (64, 65). This creates a direct mechanistic bridge between ac4C-driven metabolism and immune evasion: by stabilizing LDHA mRNA, NAT10 simultaneously accelerates the final step of glycolysis and generates the immunosuppressive metabolite that shields the tumor from cytotoxic attack. The ac4C-LDHA-lactate axis has been validated across multiple tumor contexts, in triple-negative breast cancer it operates within a broader NAT10/ac4C/JunB regulatory program (64), while in gastric cancer NAT10 directly acetylates LDHA mRNA to enhance stability and drive tumor growth (65), demonstrating that this mechanism is not context-specific but reflects a conserved post-transcriptional strategy for coupling glycolytic output to immune suppression.

### 3.4 ac4C modification of phosphoglycerate mutase 1

Phosphoglycerate mutase 1 (PGAM1) catalyzes the interconversion of 3-phosphoglycerate and 2-phosphoglycerate in glycolysis, and its overexpression in various cancers promotes tumor growth by enhancing glycolytic flux and supporting biosynthetic demands (27, 65). Beyond its canonical glycolytic role, PGAM1 has been shown to coordinate glycolysis with the pentose phosphate pathway and serine biosynthesis, making it a metabolic hub that supports multiple anabolic programs in proliferating cancer cells.

ac4C modification of PGAM1 mRNA stabilizes the transcript and enhances translation, increasing PGAM1 activity and accelerating the interconversion of 3-phosphoglycerate and 2-phosphoglycerate (66). Beyond its canonical role in glycolytic flux, this upregulation supports the biosynthetic demands of cancer stem cells, which rely heavily on glycolysis for self-renewal: NAT10 knockdown reduces both PGAM1 expression and cancer stem cell marker expression, suggesting that ac4C modification of PGAM1 mRNA contributes to the maintenance of stemness alongside metabolic reprogramming. This finding extends the functional reach of ac4C-driven glycolysis beyond energy production to encompass tumor cell plasticity and the maintenance of a therapy-resistant stem-like subpopulation.

### 3.5 The NAT10/SEPT9/HIF-1α positive feedback loop

Beyond direct modification of individual glycolytic enzyme mRNAs, ac4C modification also regulates glycolysis at a systems level through a positive feedback loop involving septin 9 (SEPT9) and hypoxia-inducible factor 1α (HIF-1α) (67–74). In gastric cancer, this loop operates as follows: HIF-1α, a master transcriptional regulator of the hypoxic response, directly activates NAT10 transcription under hypoxic conditions. NAT10 then catalyzes ac4C modification of SEPT9 mRNA, enhancing SEPT9 protein expression. SEPT9, in turn, stabilizes HIF-1α protein by preventing its proteasomal degradation, thereby completing a positive feedback circuit that sustains HIF-1α activity and glycolytic gene expression even as oxygen levels fluctuate.

This self-reinforcing NAT10/SEPT9/HIF-1α circuit has profound implications for cancer metabolism and therapy resistance. Once activated, for example by initial hypoxia or oncogenic signaling, the loop can maintain elevated glycolysis and HIF-1α activity even under normoxic conditions, contributing to the pseudo-hypoxic phenotype observed in many aggressive cancers. The loop also amplifies the expression of HIF-1α target genes beyond glycolytic enzymes, including VEGF, which drives angiogenesis, and PD-L1, which promotes immune evasion, thereby connecting metabolic reprogramming to both vascular remodeling and immune escape through a single regulatory axis.

The NAT10/SEPT9/HIF-1α feedback loop also contributes to resistance to anti-angiogenic therapy, as sustained HIF-1α activity can upregulate alternative pro-angiogenic factors when VEGF signaling is blocked (75). Pharmacological inhibition of NAT10 with Remodelin suppresses hypoxia-induced HIF-1α expression, disrupting the feedback loop and potentially restoring sensitivity to anti-angiogenic agents. This finding suggests that NAT10 inhibition may be particularly valuable in combination with anti-angiogenic therapies in tumors where the NAT10/SEPT9/HIF-1α axis is active.

### 3.6 ac4C modification in lipid metabolism

In addition to glycolysis, NAT10-mediated ac4C modification also regulates lipid metabolism in cancer cells. Unbiased RNA-seq analysis following NAT10 depletion revealed significant downregulation of genes involved in fatty acid synthesis and lipid transport, suggesting that NAT10 broadly supports lipid anabolic programs in addition to glycolysis (76). This finding is consistent with the known role of NAT10 in supporting the biosynthetic demands of rapidly proliferating cancer cells, which require both glycolytic intermediates and lipid precursors for membrane synthesis and energy storage.

Further metabolomic analysis revealed that Remodelin treatment alters mitochondrial lipid metabolism, affecting the levels of various lipid species including phosphatidylcholines, phosphatidylethanolamines, and acylcarnitines (77). These changes suggest that NAT10 inhibition disrupts the coordination between glycolysis and mitochondrial metabolism, potentially impairing the metabolic flexibility that cancer cells rely on to survive nutrient stress. The broader metabolic consequences of NAT10 inhibition, spanning glycolysis, lipid synthesis, and mitochondrial function, may contribute to its anti-tumor efficacy and warrant further investigation in the context of combination metabolic therapies.

### 3.7 Summary: ac4C as a master regulator of cancer metabolism

**Figure 3:**
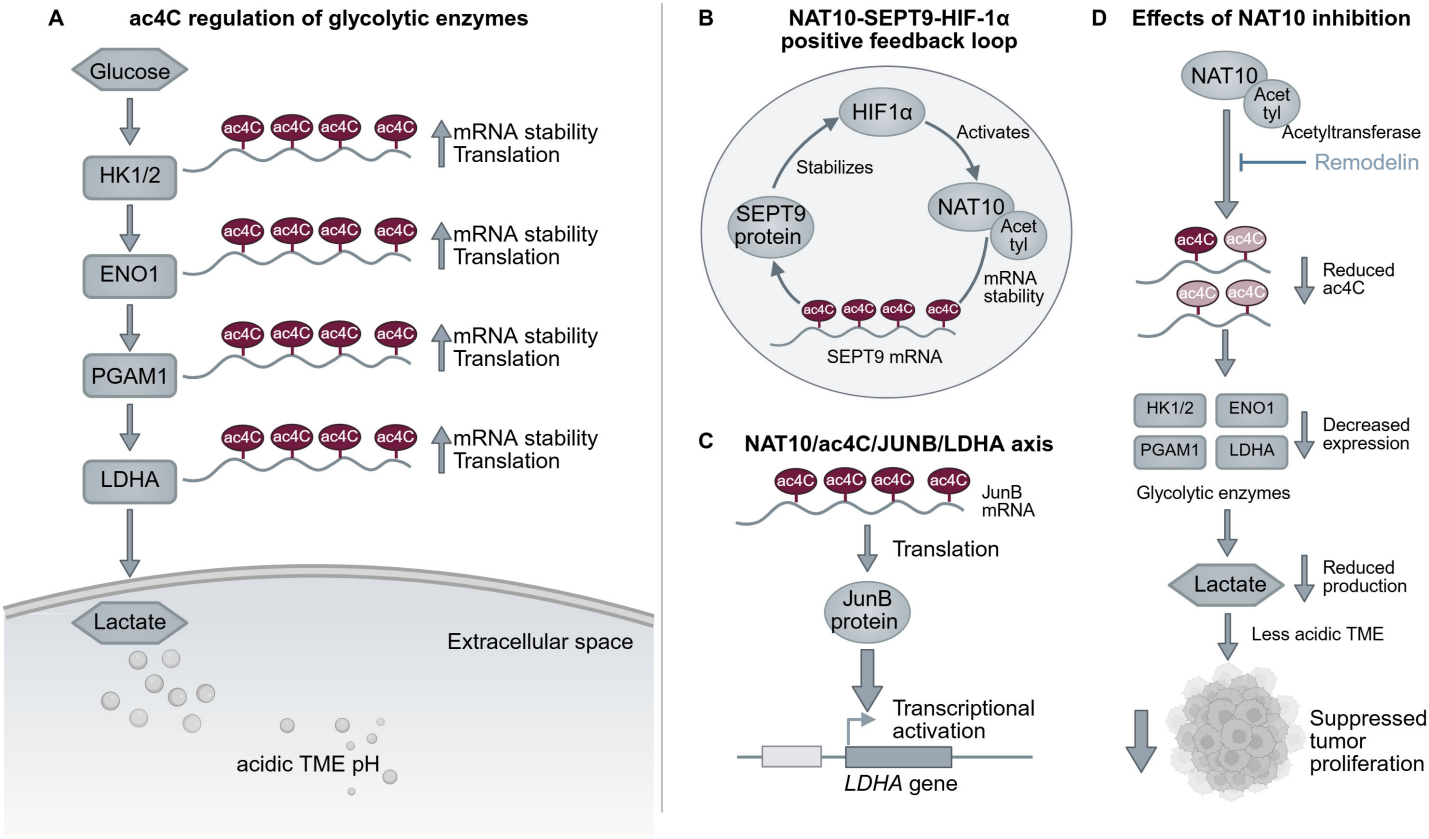
NAT10-mediated ac4C modification enhances glycolytic enzyme expression and promotes the Warburg effect. **(A)** Schematic of glycolysis pathway showing ac4C-modified enzymes. Key glycolytic enzymes regulated by ac4C modification are highlighted: HK1/2 (hexokinase, first committed step), ENO1 (enolase, penultimate step), PGAM1 (phosphoglycerate mutase), and LDHA (lactate dehydrogenase, final step). ac4C modification enhances mRNA stability and translation of these enzymes. **(B)** The NAT10/SEPT9/HIF-1α positive feedback loop. Under hypoxia, HIF-1α transcriptionally activates NAT10 expression. NAT10 catalyzes ac4C modification of SEPT9 mRNA, enhancing its stability. SEPT9 protein stabilizes HIF-1α by preventing proteasomal degradation, creating a positive feedback loop that sustains glycolytic gene expression. **(C)** The NAT10/ac4C/JunB/LDHA axis in triple-negative breast cancer. NAT10-mediated ac4C modification enhances JunB mRNA stability. JunB transcription factor directly activates LDHA transcription, leading to elevated lactate production and immunosuppressive tumor microenvironment. **(D)** Metabolic consequences of NAT10 inhibition. Remodelin treatment reduces ac4C modification levels, leading to decreased expression of glycolytic enzymes, reduced glucose uptake, diminished lactate production, and impaired tumor cell proliferation. Created with biorender.

Taken together, the studies reviewed above establish ac4C modification as a critical regulator of cancer metabolism, particularly glycolysis. By enhancing the stability and translation of key glycolytic enzymes (HK1/2, ENO1, LDHA, PGAM1) and by sustaining the NAT10/SEPT9/HIF-1α positive feedback loop, ac4C modification drives a comprehensive glycolytic reprogramming program that supports tumor growth, generates an immunosuppressive lactate-rich microenvironment, and promotes resistance to multiple therapeutic modalities. The conservation of these mechanisms across diverse cancer types, gastric, lung, breast, ovarian, retinoblastoma, suggests that ac4C-driven glycolytic reprogramming is a general feature of malignancy rather than a cancer type-specific phenomenon, strengthening the rationale for targeting NAT10 as a broadly applicable anti-cancer strategy (**Figure 3**; **Table 1**).

**Table 1:** Glycolytic enzymes regulated by ac4C modification in cancer.

Target gene	Function in glycolysis	Cancer type	Mechanism	Functional outcome
HK2	First committed step (glucose → G6P)	Gastric cancer	ac4C enhances HK2 mRNA stability	Increased glycolytic flux
HK1	First committed step (glucose → G6P)	Retinoblastoma	ac4C enhances HK1 mRNA stability	Promoted glycolysis
ENO1	Penultimate step (2-PG → PEP)	NSCLC	NAT10 binds and modifies ENO1 mRNA	Enhanced glycolysis
LDHA	Final step (pyruvate → lactate)	TNBC	ac4C of JunB → LDHA transcription	Lactate production, immunosuppression
LDHA	Final step (pyruvate → lactate)	Gastric cancer	Direct ac4C of LDHA mRNA	Enhanced LDHA stability
LDHA/PFKM	Lactate production/rate-limiting	Osteosarcoma	ac4C-m6A crosstalk via YTHDC1	Glycolysis promotion
PGAM1	3-PG ↔ 2-PG interconversion	Ovarian cancer	ac4C enhances PGAM1 mRNA stability	Glycolysis and stemness
SEPT9	HIF-1α stabilization (indirect)	Gastric cancer	ac4C of SEPT9 → HIF-1α stability	Positive feedback loop
FOXP1	Transcription factor for glycolytic genes	Cervical cancer	ac4C enhances FOXP1 mRNA stability	Glycolysis and immunosuppression

G6P, glucose-6-phosphate; 2-PG, 2-phosphoglycerate; PEP, phosphoenolpyruvate; NSCLC, non-small cell lung cancer; TNBC, triple-negative breast cancer.

## 4 ac4C modification in tumor immune evasion

Immune evasion is another hallmark of cancer, enabling tumor cells to escape surveillance and destruction by the immune system (28–31, 78, 79). Emerging evidence demonstrates that NAT10-mediated ac4C modification plays a multifaceted role in this process, operating through at least four distinct mechanisms: upregulation of immune checkpoint molecules, direct suppression of cytotoxic T cell function, inhibition of type I interferon signaling, and remodeling of the tumor microenvironment. The breadth of these immune-evasive functions, combined with the metabolic roles described in Section 3, positions NAT10 as a dual regulator of cancer hallmarks with exceptional therapeutic relevance.

### 4.1 Regulation of PD-L1 expression

Programmed death-ligand 1 (PD-L1) is a key immune checkpoint molecule that suppresses anti-tumor immunity by engaging PD-1 on T cells, triggering T cell exhaustion and apoptosis (80–84). PD-L1 overexpression in tumors is a major mechanism of immune evasion and a predictive biomarker for response to anti-PD-1/PD-L1 immunotherapy. Multiple independent studies have now demonstrated that ac4C modification regulates PD-L1 expression through distinct molecular mechanisms, establishing NAT10 as a convergence point for multiple immune checkpoint regulatory pathways.

One mechanism by which ac4C modification drives PD-L1 upregulation involves the tryptophan metabolite kynurenine, which is enriched in the tumor microenvironment as a consequence of IDO1 activity. In colorectal cancer, kynurenine promotes immune escape by activating the aryl hydrocarbon receptor (AhR), which transcriptionally upregulates NAT10 expression (85). NAT10 then catalyzes ac4C modification of PD-L1 mRNA, enhancing its stability and translation, and thereby increasing surface PD-L1 levels on tumor cells. This kynurenine-AhR-NAT10-PD-L1 axis represents a direct mechanistic link between tryptophan catabolism and immune checkpoint regulation, with important implications for the design of combination immunotherapy strategies.

An alternative mechanism of NAT10-mediated PD-L1 regulation operates through the nucleolar protein NPM1 (86). In this pathway, NAT10 acetylates NPM1 as a protein substrate, and acetylated NPM1 translocates from the nucleolus to the nucleus where it activates PD-L1 transcription. This protein acetyltransferase activity of NAT10, distinct from its RNA modification function, thus contributes to immune evasion through a transcriptional rather than post-transcriptional mechanism. Targeting the NAT10/NPM1 axis with NAT10 inhibitors abrogated PD-L1 expression and improved the response to immune checkpoint blockade therapy across multiple cancer types, demonstrating the therapeutic relevance of this pathway.

A third mechanism involves the nuclear import receptor KPNB1, which mediates PD-L1 nuclear translocation in NSCLC (87). NAT10-mediated ac4C modification enhances KPNB1 mRNA stability and translation, increasing KPNB1 protein levels and promoting the nuclear translocation of PD-L1. Nuclear PD-L1 has been shown to regulate gene transcription and promote radiotherapy resistance, suggesting that ac4C modification of KPNB1 mRNA contributes to both immune evasion and treatment resistance through a single molecular event. The existence of three distinct NAT10-dependent mechanisms for PD-L1 regulation, post-transcriptional stabilization of PD-L1 mRNA, transcriptional activation via NPM1, and nuclear translocation via KPNB1, underscores the central importance of NAT10 in immune checkpoint control.

### 4.2 Suppression of T cell function

Beyond regulating PD-L1 expression, NAT10-mediated ac4C modification directly suppresses T cell function through multiple mechanisms (80, 88, 89). The suppression of cytotoxic CD8+ T cells is particularly consequential, as these cells are the primary effectors of anti-tumor immunity and their dysfunction is a major barrier to immunotherapy efficacy.

NAT10 suppresses CD8^+^ T cell function through at least two mechanistically distinct routes. The first operates cell-autonomously within tumor cells: high NAT10 activity drives the expression of immunosuppressive factors that reduce tumor immunogenicity and promote T cell exhaustion, while NAT10 inhibition simultaneously increases tumor cell immunogenicity and restores T cell cytotoxic function, effects that synergize with anti-PD-1 therapy to achieve superior tumor control (90). The second route is indirect, mediated by secreted factors: NAT10-mediated ac4C modification stabilizes DDX5 mRNA, elevating DDX5 protein levels, which in turn promotes the secretion of HMGB1 into the tumor microenvironment (91). HMGB1 acts as an immunosuppressive alarmin that activates regulatory T cells and suppresses cytotoxic T cell responses, effectively converting the extracellular space into a T cell-hostile niche. Disrupting this axis with Remodelin reduces HMGB1 secretion and restores T cell activity, and the combination with anti-PD-1 therapy achieves synergistic tumor control demonstrating that ac4C-driven T cell suppression is pharmacologically reversible. Consistent with these mechanistic findings, high NAT10 expression in tumor tissues correlates with reduced CD8+ T cell infiltration and impaired cytotoxic function across cancer types (92), suggesting that NAT10 expression level is a reliable indicator of the immunosuppressive state of the tumor microenvironment.

### 4.3 Inhibition of type I interferon signaling

Type I interferons (IFN-I) play critical roles in anti-tumor immunity by promoting dendritic cell maturation, enhancing antigen presentation, and stimulating cytotoxic T cell responses (28, 29, 31). Tumor-intrinsic suppression of IFN-I signaling is a well-recognized mechanism of immune evasion, and recent studies have identified NAT10 as a key mediator of this suppression.

A landmark study published in Nature Communications demonstrated that inhibition of tumor-intrinsic NAT10 enhances anti-tumor immunity by triggering type I interferon responses (93). Mechanistically, NAT10 promotes the expression of MYC, which in turn activates CDK2 to phosphorylate and activate DNMT1. DNMT1-mediated DNA methylation silences the promoters of IFN-I genes and innate immune sensors, thereby suppressing the IFN-I response. NAT10 inhibition reverses this epigenetic silencing, restores IFN-I signaling, and enhances the recruitment and activation of cytotoxic T cells in the tumor microenvironment.

A complementary mechanism operates in NSCLC, where NAT10-mediated upregulation of the long non-coding RNA GAS5 facilitates immune cell infiltration through the MYBBP1A-p53/IRF1/IFN-I signaling axis (94). In this context, GAS5 acts as a scaffold that recruits MYBBP1A to the p53/IRF1 transcriptional complex, activating IFN-I gene expression and promoting the recruitment of cytotoxic immune cells. The finding that NAT10 regulates both the MYC/CDK2/DNMT1 axis and the GAS5/MYBBP1A/IRF1 axis suggests that its control of IFN-I signaling is multifaceted and may vary across cancer types and cellular contexts.

### 4.4 Remodeling the tumor microenvironment

NAT10-mediated ac4C modification also contributes to immune evasion by remodeling the tumor microenvironment (TME), affecting the composition and function of infiltrating immune cells (29, 30, 78, 95–98). The TME is a complex ecosystem comprising cancer cells, immune cells, cancer-associated fibroblasts, endothelial cells, and extracellular matrix components, and its immunosuppressive remodeling is a critical barrier to effective immunotherapy.

ac4C modification reshapes the tumor microenvironment through multiple effector mechanisms that collectively reduce immune cell infiltration and impair anti-tumor function. One route operates through GLMP mRNA stabilization: ac4C modification by NAT10 increases GLMP expression, which activates MAPK/ERK signaling in tumor cells and drives the secretion of immunosuppressive cytokines and chemokines that remodel immune cell composition in the TME (99). A second, cell-type-specific route reveals an important complexity: whereas NAT10 activity in tumor cells promotes immune evasion, downregulation of ac4C modification specifically in myeloid cells attenuates their anti-tumor function and exacerbates disease progression (100). This opposing directionality, pro-tumorigenic in cancer cells, pro-immunogenic in myeloid cells, means that systemic NAT10 inhibition could partially offset its own therapeutic benefit by impairing myeloid cell function, and underscores the need for tumor cell-targeted delivery strategies. At the population level, tumors with high NAT10 activity display reduced cytotoxic T cell infiltration and increased regulatory T cell abundance, and this immunosuppressive signature differs between microsatellite-stable and microsatellite-instable tumors (101), suggesting that the immune consequences of ac4C modification are modulated by the broader mutational landscape of the tumor.

### 4.5 Cell-type specificity of ac4C modification in the tumor microenvironment

A critical and underappreciated dimension of ac4C biology is its cell-type-specific function within the tumor microenvironment. Emerging evidence indicates that NAT10 expression levels, ac4C target gene profiles, and downstream signaling consequences differ substantially between tumor cells, myeloid cells, and T lymphocytes, with important implications for therapeutic targeting. In tumor cells, high NAT10 activity drives the pro-tumorigenic programs described throughout this review, glycolytic reprogramming, PD-L1 upregulation, and immune evasion. However, a landmark study in hepatocellular carcinoma demonstrated that ac4C modification in myeloid cells plays a diametrically opposite role: downregulation of ac4C in tumor-associated myeloid cells attenuated their anti-tumor immunostimulatory function, exacerbating HCC progression and reducing the efficacy of immunotherapy. This finding reveals a fundamental paradox for systemic NAT10 inhibition: while suppressing NAT10 in tumor cells is therapeutically desirable, simultaneously suppressing NAT10 in myeloid cells may be counterproductive by impairing their anti-tumor activity. In T cells, NAT10-mediated ac4C modification has been reported to regulate the expression of key effector molecules and exhaustion markers, though the precise ac4C target gene landscape in T cells remains incompletely characterized. The advent of single-cell ac4C profiling technologies, combined with scRNA-seq, now enables systematic dissection of NAT10 expression and ac4C target gene profiles across individual cell types within the TME. Such analyses will be essential for understanding the cell-type-specific upstream signaling pathways that regulate NAT10 activity, for example, whether oncogenic signaling (e.g., MYC, PI3K/AKT) drives NAT10 upregulation specifically in tumor cells, while cytokine signaling (e.g., IFN-γ, IL-4) modulates NAT10 in immune cells. Resolving these cell-type-specific regulatory circuits is a prerequisite for designing targeted therapeutic strategies that selectively inhibit NAT10 in malignant cells while preserving its function in anti-tumor immune cells (93, 102, 103).

### 4.6 ac4C modification of non-coding RNAs in tumor metabolic-immune crosstalk

While the preceding sections have focused on ac4C modification of protein-coding mRNAs, emerging evidence indicates that NAT10-mediated ac4C modification also targets non-coding RNAs (ncRNAs), including long non-coding RNAs (lncRNAs), circular RNAs (circRNAs), and microRNAs (miRNAs), expanding the regulatory reach of ac4C modification in cancer. Long non-coding RNAs represent a particularly important class of ac4C targets. As noted in Section 4.3, NAT10-mediated upregulation of the lncRNA GAS5 in NSCLC facilitates immune cell infiltration through the MYBBP1A-p53/IRF1/IFN-I signaling axis, illustrating how ac4C modification of lncRNA-associated regulatory networks can shape anti-tumor immunity. More broadly, ac4C modification may regulate lncRNA stability and secondary structure, thereby modulating lncRNA-target gene binding efficiency and lncRNA-mediated chromatin remodeling. Circular RNAs, which are resistant to exonucleolytic degradation and serve as miRNA sponges and protein scaffolds, may also be subject to ac4C modification, potentially altering their stability, translation (for cap-independent translation-competent circRNAs), and interaction with RNA-binding proteins. ac4C modification of circRNAs in the context of tumor metabolism and immunity remains an open area of investigation. With respect to miRNAs, ac4C modification may influence miRNA biogenesis and processing: acetylation of cytidine residues in pri-miRNA or pre-miRNA sequences could alter the recognition and cleavage efficiency of the Drosha/DGCR8 and Dicer complexes, thereby modulating the abundance of mature miRNAs that regulate metabolic and immune gene expression. Systematic profiling of ac4C modification across the non-coding transcriptome, enabled by emerging single-cell and spatial ac4C sequencing technologies, will be essential for fully mapping the ac4C regulatory network in cancer (104, 105).

### 4.7 Summary: ac4C as a multifaceted regulator of tumor immunity

**Figure 4:**
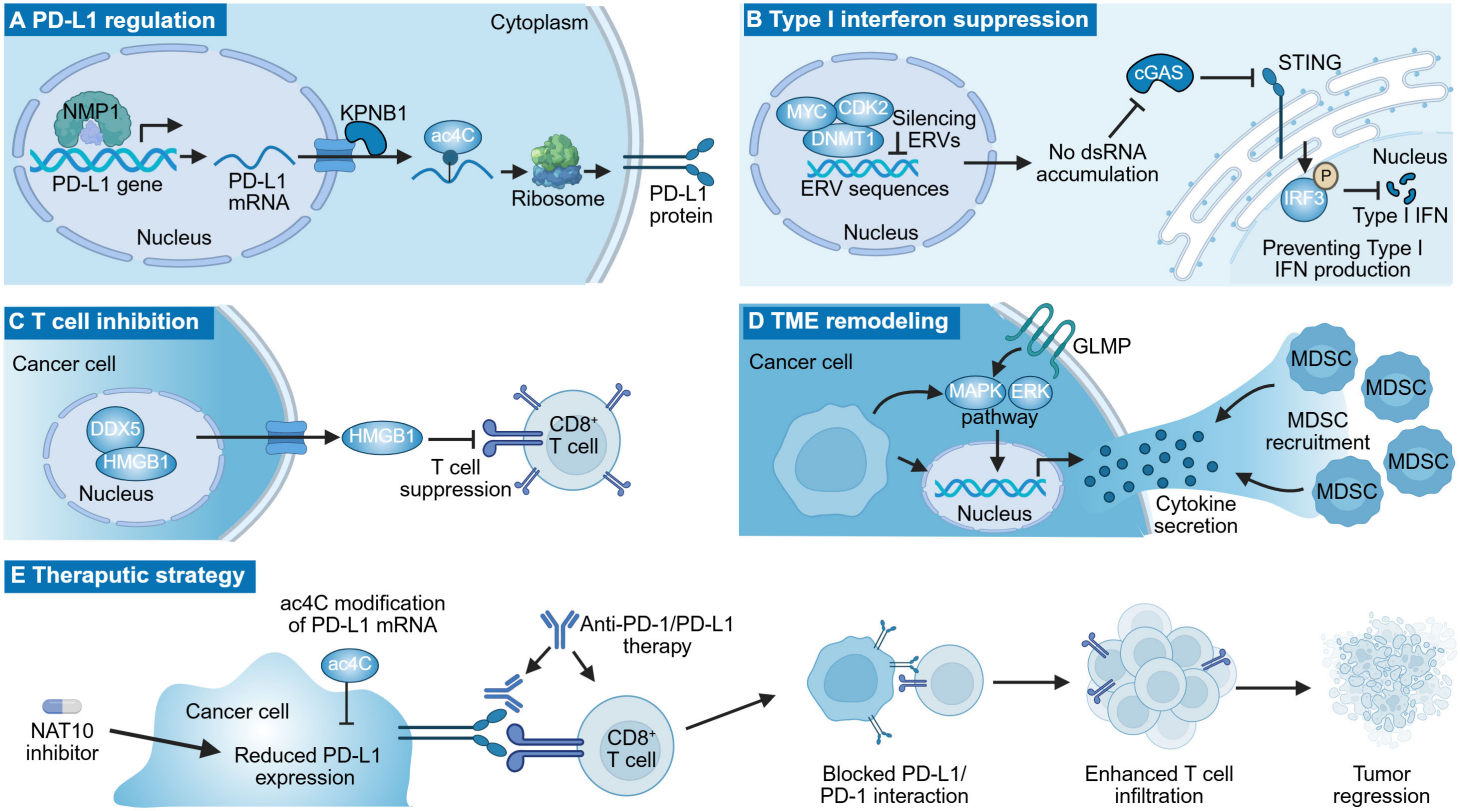
Multiple mechanisms by which ac4C modification promotes tumor immune evasion. **(A)** Regulation of PD-L1 expression by ac4C modification. Three mechanisms are illustrated: (1) Direct ac4C modification of PD-L1 mRNA enhances its stability (kynurenine-AhR-NAT10 axis); (2) NAT10 acetylates NPM1, which translocates to nucleus and activates PD-L1 transcription; (3) ac4C modification of KPNB1 mRNA promotes PD-L1 nuclear translocation and radioresistance. **(B)** Suppression of type I interferon signaling. NAT10 promotes MYC expression, which activates CDK2 and subsequently DNMT1. DNMT1-mediated DNA methylation silences endogenous retroviral elements (ERVs), preventing activation of the cGAS-STING pathway and type I interferon production. NAT10 inhibition leads to ERV derepression and robust IFN-I response. **(C)** Inhibition of T cell function. NAT10-mediated ac4C modification of DDX5 mRNA enhances DDX5 expression, which promotes HMGB1 secretion. Extracellular HMGB1 paradoxically suppresses T cell function in the tumor microenvironment. **(D)** Remodeling of tumor microenvironment. ac4C modification of GLMP mRNA activates MAPK/ERK signaling, leading to secretion of immunosuppressive cytokines, recruitment of MDSCs, and exclusion of cytotoxic T lymphocytes. **(E)** Therapeutic implications. Combination of NAT10 inhibitors (Remodelin) with anti-PD-1/PD-L1 antibodies shows synergistic anti-tumor effects by simultaneously reducing PD-L1 expression and enhancing T cell infiltration and function. Created with biorender.

The studies reviewed above establish ac4C modification as a multifaceted regulator of tumor immunity, operating through at least four distinct mechanisms: upregulation of PD-L1 expression (via direct mRNA stabilization, NPM1-mediated transcription, and KPNB1-mediated nuclear translocation), direct suppression of CD8+ T cell function, inhibition of type I interferon signaling (via MYC/CDK2/DNMT1 and GAS5/MYBBP1A/IRF1 axes), and remodeling of the tumor microenvironment. The convergence of these mechanisms on NAT10 as a common upstream regulator suggests that targeting this single enzyme could simultaneously reverse multiple immune evasion strategies, potentially achieve broader immunological effects than target any individual downstream pathway. However, the cell type-specific effects of ac4C modification, particularly the opposing roles in tumor cells versus myeloid cells, highlight the need for tumor cell-targeted delivery strategies to maximize therapeutic benefit (**Figure 4**; **Table 2**).

**Table 2:** Immune-related targets of ac4C modification in cancer.

Target/pathway	Immune function	Cancer type	Mechanism	Functional outcome
PD-L1 (direct)	Immune checkpoint	Colorectal cancer	Kynurenine→AhR→NAT10→ac4C of PD-L1	Enhanced PD-L1, T cell suppression
PD-L1 (via NPM1)	Immune checkpoint	Multiple cancers	NAT10 acetylates NPM1→PD-L1 transcription	Improved ICI response upon inhibition
PD-L1 (via KPNB1)	Nuclear PD-L1, DNA repair	NSCLC	ac4C of KPNB1→PD-L1 nuclear translocation	Radioresistance, immune evasion
Type I IFN	Anti-tumor immunity	Multiple cancers	NAT10→MYC→CDK2→DNMT1→ERV silencing	IFN-I suppression
GAS5/IFN-I	Immune cell infiltration	NSCLC	ac4C of GAS5→MYBBP1A-p53/IRF1/IFN-I	Context-dependent effects
DDX5/HMGB1	T cell suppression	NPC	ac4C of DDX5→HMGB1 secretion	T cell dysfunction
CD8+ T cells	Cytotoxic function	Pancreatic cancer	Immunosuppressive factor secretion	Improved T cell function upon inhibition
CD8+ T cells	T cell infiltration	Prostate cancer	Immunosuppressive cytokines	Reduced infiltration, poor prognosis
GLMP/MAPK/ERK	TME remodeling	HNSCC	ac4C of GLMP→MAPK/ERK activation	MDSC recruitment, CTL exclusion
Myeloid ac4C	Cell type-specific	HCC	Myeloid ac4C suppresses PD-L1	Remodelin attenuates ICI efficacy

ICI, immune checkpoint inhibitor; NSCLC, non-small cell lung cancer; NPC, nasopharyngeal carcinoma; HNSCC, head and neck squamous cell carcinoma; HCC, hepatocellular carcinoma; TME, tumor microenvironment; MDSC, myeloid-derived suppressor cell; CTL, cytotoxic T lymphocyte.

## 5 Metabolic-immune crosstalk mediated by ac4C modification

The preceding sections have separately discussed the roles of ac4C modification in tumor metabolism and immune evasion. However, these two processes are not independent but are intricately interconnected, with metabolic reprogramming directly shaping the immune landscape of the tumor microenvironment and immune signals in turn regulating metabolic programs (96, 106–108). ac4C modification sits at the intersection of these two hallmarks, functioning as a molecular bridge that coordinates metabolic and immunological programs to create a self-reinforcing pro-tumorigenic state. This section synthesizes the evidence for this metabolic-immune crosstalk and proposes an integrated model of ac4C function in cancer.

### 5.1 Glycolysis-driven immunosuppression: the lactate connection

The Warburg effect, characterized by enhanced glycolysis and lactate production, creates an acidic and immunosuppressive tumor microenvironment that directly impairs anti-tumor immunity (109–116). Lactate, once considered merely a metabolic waste product, is now recognized as a potent immunomodulatory molecule that shapes the function of virtually every immune cell type in the TME. The immunosuppressive consequences of lactate accumulation are therefore a direct downstream consequence of the ac4C-driven glycolytic program described in Section 3, establishing a mechanistic link between NAT10 activity and immune evasion that operates through metabolic intermediates rather than direct regulation of immune genes.

Lactate suppresses anti-tumor immunity through multiple complementary mechanisms (117–123). High lactate concentrations impair T cell receptor signaling, reduce the production of effector cytokines including IFN-γ and TNF-α, and promote T cell exhaustion by enforcing MCT11-mediated lactate uptake that disrupts mitochondrial metabolism. Simultaneously, lactate promotes the differentiation and stability of immunosuppressive regulatory T cells (Tregs), which use lactate as a metabolic fuel and are paradoxically enhanced by the acidic conditions that impair conventional T cells. Lactate also suppresses natural killer (NK) cell cytotoxicity and promotes the polarization of tumor-associated macrophages toward an immunosuppressive M2 phenotype, collectively creating a TME that is hostile to anti-tumor immunity.

The studies reviewed in Section 3 demonstrate that NAT10-mediated ac4C modification enhances the expression of key glycolytic enzymes, particularly LDHA, which catalyzes lactate production. In TNBC, NAT10-driven LDHA upregulation increases lactate secretion, creating an acidic microenvironment that impairs CD8+ T cell function (64). This finding directly connects the metabolic and immune functions of NAT10: by enhancing LDHA expression through ac4C modification, NAT10 simultaneously accelerates glycolysis and generates the lactate that suppresses anti-tumor immunity, achieving dual pro-tumorigenic effects through a single post-transcriptional regulatory event.

### 5.2 The NAT10/ac4C/FOXP1 axis: a paradigm of metabolic-immune integration

The most direct evidence for ac4C-mediated metabolic-immune integration comes from a study in cervical cancer demonstrating that NAT10-mediated ac4C modification of FOXP1 mRNA simultaneously drives glycolytic reprogramming and immunosuppression (124). FOXP1, a forkhead box transcription factor, activates the transcription of glycolytic genes while also suppressing the expression of immunostimulatory molecules, thereby linking metabolic and immune programs at the transcriptional level. NAT10-mediated ac4C modification enhances FOXP1 mRNA stability and translation, increasing FOXP1 protein levels and activating both its metabolic and immunosuppressive transcriptional programs. This study established the NAT10/ac4C/FOXP1 axis as a paradigm of metabolic-immune integration, in which a single RNA modification event coordinates two cancer hallmarks through a shared transcriptional effector.

Mechanistically, FOXP1 activates the transcription of glycolytic genes including HK2, LDHA, and PKM2, promoting the Warburg effect and lactate production. Simultaneously, FOXP1 suppresses the expression of MHC class I molecules and co-stimulatory ligands on tumor cells, reducing their immunogenicity and impairing T cell recognition. The dual transcriptional activity of FOXP1 thus creates a state in which tumor cells are simultaneously metabolically hyperactive and immunologically invisible, a combination that is particularly resistant to both metabolic and immunological therapeutic interventions when applied individually.

The glycolytic phenotype induced by FOXP1 further contributes to immunosuppression through lactate-mediated mechanisms, creating a vicious cycle in which ac4C modification drives glycolysis, glycolysis generates lactate, and lactate suppresses the immune cells that would otherwise eliminate the tumor. This cycle is self-reinforcing because the immunosuppressive TME created by lactate reduces immune pressure on tumor cells, allowing them to continue proliferating and generating more lactate.

Targeting NAT10 with Remodelin disrupted this vicious cycle, reducing both glycolysis and immunosuppression in cervical cancer models (124). Combination of Remodelin with anti-PD-1 therapy showed synergistic anti-tumor effects, providing proof-of-concept that simultaneously targeting the metabolic and immune arms of the NAT10/ac4C/FOXP1 axis can achieve superior anti-tumor efficacy compared to either approach alone. This study provides the most compelling evidence to date that ac4C modification functions as a molecular bridge between metabolic reprogramming and immune evasion, and that targeting this bridge can simultaneously reverse both cancer hallmarks.

### 5.3 Kynurenine-ac4C-PD-L1 axis: metabolite-driven immune checkpoint regulation

The tryptophan-kynurenine pathway represents another mechanism by which tumor metabolism directly regulates ac4C-mediated immune checkpoint expression (85, 125–138). IDO1-mediated tryptophan catabolism generates kynurenine, which accumulates in the TME and activates the aryl hydrocarbon receptor (AhR) in both tumor cells and immune cells. In tumor cells, AhR activation transcriptionally upregulates NAT10 expression, which then catalyzes ac4C modification of PD-L1 mRNA, enhancing its stability and translation. The resulting increase in surface PD-L1 levels suppresses T cell function through the PD-1/PD-L1 checkpoint, completing a metabolic-immune regulatory circuit that connects tryptophan catabolism to immune checkpoint upregulation through ac4C modification.

This kynurenine-AhR-NAT10-PD-L1 axis operates in colorectal cancer and likely in other tumor types where IDO1 is active, given the conservation of AhR signaling and NAT10 expression across cancer types (85). The axis is particularly significant because it provides a mechanistic explanation for the observation that tumors with high IDO1 activity are often resistant to anti-PD-1 therapy: IDO1-generated kynurenine not only directly suppresses T cells but also upregulates PD-L1 through NAT10, creating a dual immunosuppressive mechanism that may overwhelm the effects of PD-1 blockade alone.

This kynurenine-ac4C-PD-L1 axis has important therapeutic implications. Targeting IDO1 with inhibitors such as epacadostat reduces kynurenine production, which should reduce AhR activation and NAT10-mediated PD-L1 upregulation. However, clinical trials of IDO1 inhibitors combined with anti-PD-1 therapy have shown disappointing results, possibly because kynurenine-independent mechanisms of PD-L1 regulation compensate for IDO1 inhibition. Targeting NAT10 downstream of AhR may therefore be a more effective strategy for blocking kynurenine-driven immune checkpoint upregulation, particularly in combination with IDO1 inhibitors and anti-PD-L1 antibodies.

### 5.4 HIF-1α as a central hub of metabolic-immune crosstalk

Hypoxia-inducible factor 1α (HIF-1α) is a master regulator of both metabolic reprogramming and immune evasion, making it a central hub of metabolic-immune crosstalk (68–74, 139–141). Under hypoxic conditions, HIF-1α activates the transcription of glycolytic genes, promoting the Warburg effect, while simultaneously upregulating PD-L1 expression and suppressing the expression of NK cell-activating ligands, thereby coordinating metabolic adaptation with immune evasion. The NAT10/SEPT9/HIF-1α positive feedback loop described in Section 3.5 thus has dual metabolic and immunological consequences: it sustains glycolytic enzyme expression while also maintaining HIF-1α-driven immune checkpoint upregulation, creating a self-reinforcing state of metabolic-immune co-adaptation.

The NAT10/SEPT9/HIF-1α feedback loop identified in gastric cancer (67) has important immunological implications beyond its metabolic effects. HIF-1α directly activates PD-L1 transcription through hypoxia response elements in the PD-L1 promoter, and sustained HIF-1α activity driven by the NAT10/SEPT9 loop would therefore maintain elevated PD-L1 expression even under normoxic conditions. This pseudo-hypoxic immune evasion, where HIF-1α-driven PD-L1 upregulation persists in the absence of oxygen deprivation, may explain why some tumors with high NAT10 expression are resistant to anti-PD-1 therapy despite adequate tumor oxygenation.

Remodelin-mediated suppression of HIF-1α expression (75) therefore has dual therapeutic consequences: it disrupts the metabolic arm of the NAT10/SEPT9/HIF-1α loop by reducing glycolytic enzyme transcription, and it disrupts the immune arm by reducing HIF-1α-driven PD-L1 upregulation. This dual effect provides a mechanistic rationale for combining NAT10 inhibitors with anti-angiogenic agents and immune checkpoint inhibitors, as targeting NAT10 may simultaneously reverse the metabolic and immunological consequences of HIF-1α hyperactivation.

## 6 Integrated model: ac4C as a molecular bridge

Based on the evidence reviewed above, we propose an integrated model in which ac4C modification serves as a molecular bridge connecting tumor metabolism to immune evasion (**Figure 1**). This model synthesizes the individual mechanistic findings into a coherent framework that explains how a single RNA modification enzyme can simultaneously drive two cancer hallmarks and why targeting NAT10 may achieve broader anti-tumor effects than targeting either hallmark individually.

In this model, NAT10-mediated ac4C modification enhances the stability and translation of key glycolytic enzymes (HK1/2, ENO1, LDHA, PGAM1), promoting the Warburg effect and lactate production. The resulting lactate-rich, acidic microenvironment directly suppresses cytotoxic T cell and NK cell function, impairs dendritic cell maturation, and promotes Treg expansion, creating an immunosuppressive TME. Simultaneously, ac4C modification regulates immune checkpoint molecules (PD-L1 via direct mRNA stabilization, NPM1-mediated transcription, and KPNB1-mediated nuclear translocation), suppresses type I interferon signaling (via MYC/CDK2/DNMT1 and GAS5/MYBBP1A/IRF1 axes), and promotes the secretion of immunosuppressive factors (via DDX5/HMGB1 and GLMP/MAPK/ERK pathways).

Critically, metabolic and immunological pathways are interconnected through several molecular nodes that amplify the pro-tumorigenic effects of NAT10 activity. The FOXP1 transcription factor, whose mRNA is stabilized by ac4C modification, simultaneously activates glycolytic gene transcription and suppresses immunostimulatory molecule expression, directly coupling metabolic and immune programs. The kynurenine-AhR-NAT10 axis connects tryptophan catabolism to PD-L1 upregulation through ac4C modification, linking tumor metabolism to immune checkpoint regulation. The NAT10/SEPT9/HIF-1α feedback loop sustains both glycolytic enzyme expression and HIF-1α-driven PD-L1 upregulation, creating a self-reinforcing state of metabolic-immune co-adaptation. Glycolysis-derived lactate directly suppresses anti-tumor immunity while also promoting Treg expansion, further amplifying the immunosuppressive consequences of ac4C-driven glycolysis.

This integrated model suggests that targeting NAT10/ac4C modification may simultaneously reverse metabolic reprogramming and enhance anti-tumor immunity, providing a strong rationale for combination therapies that exploit the dual metabolic-immune functions of NAT10. The model also predicts that tumors with high NAT10 expression and active metabolic-immune crosstalk may be particularly responsive to NAT10 inhibition, while tumors with low NAT10 expression or alternative metabolic-immune regulatory mechanisms may require different therapeutic approaches.

## 7 Therapeutic implications of metabolic-immune crosstalk

The metabolic-immune crosstalk mediated by ac4C modification has important therapeutic implications that extend beyond the direct anti-tumor effects of NAT10 inhibition. Because NAT10 simultaneously drives metabolic reprogramming and immune evasion, its inhibition may achieve synergistic anti-tumor effects by simultaneously reversing both cancer hallmarks, an effect that cannot be achieved by targeting either hallmark individually. This dual-targeting potential positions NAT10 inhibitors as uniquely valuable agents in the cancer therapeutic armamentarium, particularly in combination with immunotherapy.

The interconnection between metabolism and immunity mediated by NAT10 suggests several specific combination strategies. NAT10 inhibitors may be combined with metabolic modulators such as glycolysis inhibitors (e.g., 2-DG, lonidamine) or IDO inhibitors to simultaneously target multiple nodes of the metabolic-immune crosstalk network. The combination of NAT10 inhibitors with immune checkpoint inhibitors is particularly well-supported by preclinical evidence, as NAT10 inhibition reduces PD-L1 expression while simultaneously reversing the immunosuppressive metabolic microenvironment, potentially achieving synergistic immunological effects.

NAT10 expression and ac4C modification levels may also serve as biomarkers for predicting response to immunotherapy and identifying patients who would benefit from combination therapies. Tumors with high NAT10 expression are likely to have both elevated PD-L1 levels and an immunosuppressive metabolic microenvironment, making them candidates for combination NAT10 inhibitor plus immune checkpoint inhibitor therapy. Conversely, tumors with low NAT10 expression may not benefit from NAT10-targeted approaches and may require alternative strategies.

Metabolic reprogramming is a major mechanism of resistance to immunotherapy, as the lactate-rich, acidic TME created by glycolysis impairs the function of adoptively transferred T cells and reduces the efficacy of checkpoint inhibitors. Targeting ac4C modification may overcome this resistance by reversing the immunosuppressive metabolic microenvironment, restoring T cell function, and enhancing the efficacy of co-administered immunotherapies. This rationale is supported by preclinical studies demonstrating that Remodelin treatment reduces lactate production, improves T cell infiltration, and synergizes with anti-PD-1 therapy in multiple cancer models.

## 8 Crosstalk between ac4C and other RNA modifications

RNA modifications do not function in isolation but form a complex regulatory network in which different modifications influence each other’s deposition, recognition, and functional consequences (1, 3–7, 142). Recent studies have revealed important crosstalk between ac4C and other RNA modifications, particularly m6A, which is the most abundant internal mRNA modification and has been extensively studied in cancer. Understanding this crosstalk is essential for predicting the full consequences of targeting individual RNA modification enzymes and for designing rational combination therapies that exploit epitranscriptomic vulnerabilities.

### 8.1 ac4C-m6A crosstalk in cancer

A novel crosstalk between ac4C and m6A modifications was discovered in osteosarcoma, where NAT10-mediated ac4C modification was found to regulate global m6A levels through an indirect mechanism (11, 143–151). NAT10 knockdown enhanced global m6A levels in osteosarcoma cells, and further investigation revealed that NAT10 regulates the expression of m6A writers and erasers, thereby modulating the overall m6A landscape. Specifically, ac4C modification of YTHDC1 mRNA, a key m6A reader, and of LDHA and PFKM mRNAs was found to regulate glycolysis through an m6A-dependent mechanism, establishing a direct link between ac4C modification, m6A reading, and metabolic reprogramming.

The mechanistic basis of this ac4C-m6A crosstalk involves the regulation of m6A writer and eraser expression by NAT10-mediated ac4C modification of their mRNAs. When NAT10 is active, ac4C modification stabilizes the mRNAs of specific m6A regulators, maintaining a particular m6A landscape that supports glycolysis and tumor growth. When NAT10 is inhibited, the destabilization of these mRNAs shifts the m6A landscape, with downstream consequences for the expression of metabolic and immune genes that are regulated by m6A. This finding suggests that the transcriptome-wide consequences of NAT10 inhibition extend beyond the direct loss of ac4C modification to include secondary changes in m6A patterns, potentially amplifying the anti-tumor effects of NAT10-targeted therapies.

The directionality of this ac4C-m6A crosstalk warrants careful mechanistic consideration. The observation that NAT10 knockdown increases global m6A levels in osteosarcoma implies that active NAT10/ac4C signaling normally suppresses m6A deposition or promotes m6A removal. Two non-mutually exclusive mechanisms may explain this inverse relationship. First, NAT10-mediated ac4C modification may stabilize the mRNA of m6A eraser proteins such as FTO (fat mass and obesity-associated protein) or ALKBH5, thereby maintaining active m6A demethylation and keeping global m6A levels low. Under this model, NAT10 knockdown would destabilize FTO/ALKBH5 mRNA, reducing eraser activity and allowing m6A to accumulate. Second, ac4C modification may destabilize the mRNAs of m6A writer complex components such as METTL3 or METTL14, thereby suppressing m6A deposition. Under this model, NAT10 knockdown would paradoxically stabilize METTL3/METTL14 mRNA (by relieving ac4C-mediated destabilization), increasing writer activity and global m6A levels. Distinguishing between these models requires direct measurement of FTO, ALKBH5, METTL3, and METTL14 protein levels and enzymatic activities following NAT10 manipulation, combined with ac4C site mapping on their respective mRNAs. Clarifying this mechanism is important because it determines whether the therapeutic effects of NAT10 inhibition are mediated in part through m6A pathway remodeling, and whether co-targeting both ac4C and m6A pathways would produce additive or antagonistic effects (103, 152).

This study has important implications for understanding the complexity of epitranscriptomic regulation in cancer. It suggests that targeting a single RNA modification enzyme may have broader effects on the epitranscriptome than anticipated, as changes in one modification can cascade to affect others through regulatory crosstalk. This complexity must be considered when interpreting the results of NAT10 inhibition studies and when designing combination therapies targeting multiple RNA modification pathways.

### 8.2 Implications for combination therapies

The crosstalk between ac4C and m6A modifications suggests that combination therapies targeting multiple RNA modifications may be more effective than single-agent approaches (8, 9, 153–160). For example, combining NAT10 inhibitors with inhibitors of m6A writers (e.g., METTL3 inhibitors) or m6A readers (e.g., YTHDF inhibitors) may achieve synergistic effects by simultaneously disrupting multiple post-transcriptional regulatory programs that support tumor growth and immune evasion. However, the complexity of epitranscriptomic crosstalk also raises concerns about potential toxicity and off-target effects, as disrupting multiple RNA modification pathways simultaneously may have unpredictable consequences for normal cell function.

Further studies are needed to fully characterize the interactions between different RNA modifications in cancer and to identify the optimal combinations and sequences of epitranscriptomic targeting strategies. Single-cell epitranscriptomic approaches that can simultaneously profile multiple RNA modifications in individual cells will be particularly valuable for understanding how ac4C-m6A crosstalk varies across cell types within the tumor microenvironment.

### 8.3 Crosstalk between ac4C and other RNA modifications: m5C, pseudouridine, and m6Am

Beyond the ac4C-m6A axis, ac4C modification is likely to engage in regulatory crosstalk with other prevalent RNA modifications, including 5-methylcytidine (m5C), pseudouridine (Ψ), and N6,2′-O-dimethyladenosine (m6Am). 5-methylcytidine (m5C) is deposited on mRNA by NSUN2 and TRDMT1, and like ac4C, m5C modification enhances mRNA stability and translational efficiency. Both m5C and ac4C target cytidine residues, raising the possibility of competitive or cooperative modification at shared sites. In cancer, m5C modification has been shown to regulate immune evasion by stabilizing PD-L1 mRNA and modulating the tumor microenvironment, functions that parallel those of ac4C. Whether ac4C and m5C co-occur on the same transcripts, and whether they synergistically or antagonistically regulate mRNA fate, remains to be determined. Pseudouridine (Ψ), the most abundant internal RNA modification, is deposited by pseudouridine synthases (PUSs) and stabilizes RNA secondary structure by enhancing base stacking. Ψ modification of mRNA has been shown to reduce innate immune sensing by decreasing recognition by pattern recognition receptors such as TLR3 and TLR7, a function that may intersect with ac4C-mediated suppression of type I interferon signaling. Crosstalk between Ψ and ac4C in regulating mRNA immunogenicity and translational efficiency in cancer cells represents an unexplored but potentially important regulatory axis. N6,2′-O-dimethyladenosine (m6Am) is deposited at the first transcribed nucleotide adjacent to the 5′ cap by PCIF1, and stabilizes mRNA against decapping and degradation. m6Am modification has been linked to regulation of mRNA stability in cancer, and its interplay with ac4C, which also enhances mRNA stability, may create additive or redundant stabilization of oncogenic transcripts. Systematic co-mapping of ac4C with m5C, Ψ, and m6Am using multi-modification sequencing approaches will be essential for understanding the combinatorial epitranscriptomic code that governs mRNA fate in cancer cells and immune cells within the tumor microenvironment (161).

## 9 Therapeutic strategies targeting ac4C modification

The critical roles of NAT10-mediated ac4C modification in cancer metabolism and immunity make it an attractive therapeutic target, and several strategies for targeting this axis are currently under investigation (33, 35, 37, 38, 162). These range from direct pharmacological inhibition of NAT10 enzymatic activity to combination strategies that exploit the metabolic and immunological vulnerabilities created by NAT10 inhibition, and to the use of NAT10 expression as a prognostic and predictive biomarker.

### 9.1 Remodelin: the prototype NAT10 inhibitor

Remodelin (4-(4-cyanophenyl)-2-(2-cyclopentylidenehydrazinyl)thiazole) is a small molecule inhibitor of NAT10 that was originally developed for the treatment of Hutchinson-Gilford progeria syndrome (HGPS), a premature aging disorder caused by mutations in lamin A (77, 163). Remodelin inhibits NAT10 acetyltransferase activity by competing with acetyl-CoA binding, thereby reducing both RNA and protein acetylation by NAT10. Its repurposing for cancer therapy was motivated by the observation that NAT10 is frequently overexpressed in cancer and that its inhibition reduces tumor cell proliferation, migration, and invasion across multiple cancer types.

Multiple preclinical studies have demonstrated the anti-tumor efficacy of Remodelin across various cancer types, establishing it as a broadly active NAT10 inhibitor with potential for clinical translation.

Across preclinical models, Remodelin exerts anti-tumor activity through several converging mechanisms. The most consistently observed effect is reversal of epithelial-mesenchymal transition (EMT): Remodelin reduces the expression of mesenchymal markers, restores epithelial identity, and impairs cell migration and invasion (164, 165). This anti-EMT activity translates directly into chemosensitization, doxorubicin resistance in breast cancer and HCC, which is driven by NAT10-dependent EMT, is reversed by Remodelin, and the combination achieves synergistic anti-tumor effects (165). Beyond EMT, Remodelin impairs DNA replication in a manner that is independent of androgen receptor signaling, conferring activity across both castration-sensitive and castration-resistant disease states (166). In HCC, Remodelin additionally suppresses ER stress-mediated metastasis and lenvatinib resistance by reducing ac4C modification of HSP90AA1 mRNA (167), illustrating that the therapeutic consequences of NAT10 inhibition extend to multiple resistance mechanisms operating through distinct molecular intermediates. Collectively, these findings establish Remodelin as a broadly active agent whose efficacy is not confined to a single cancer type or resistance mechanism, but rather reflects the pleiotropic consequences of disrupting NAT10-mediated post-transcriptional regulation.

### 9.2 Combination with immune checkpoint inhibitors

Given the role of ac4C modification in immune evasion, combining NAT10 inhibitors with immune checkpoint inhibitors (ICIs) represents a particularly promising therapeutic strategy, as NAT10 inhibition may simultaneously reduce PD-L1 expression, reverse the immunosuppressive metabolic microenvironment, and restore T cell function.

Several preclinical studies have demonstrated synergistic effects of this combination across multiple cancer types. 1) The mechanistic rationale for this combination is strong: NAT10 inhibition simultaneously reduces PD-L1 expression through multiple pathways, reverses the immunosuppressive metabolic microenvironment by reducing lactate production, and restores CD8+ T cell cytotoxic function, three complementary effects that address distinct barriers to immunotherapy efficacy. 2) Preclinical evidence confirms synergy: Remodelin combined with anti-PD-1 therapy achieves superior tumor control compared to either agent alone, with the combination working through dual modulation of tumor cell immunogenicity and T cell function (90, 91). Targeting the NAT10/NPM1 axis, which abrogates PD-L1 expression at the transcriptional level, similarly improves the response to immune checkpoint blockade across multiple tumor types (86), suggesting that the synergy between NAT10 inhibition and ICI therapy is not dependent on a single downstream mechanism but reflects the broad immunological consequences of disrupting NAT10 activity.

### 9.3 Combination with other targeted therapies

NAT10 inhibitors may also be combined with other targeted therapies to achieve synergistic effects, exploiting the multiple vulnerabilities created by NAT10 inhibition.

In colorectal cancer, EGFR inhibition augments the therapeutic efficacy of Remodelin, and the combination of Remodelin with cetuximab showed enhanced anti-tumor effects compared to either agent alone (168). This synergy may reflect the complementary mechanisms of the two agents: EGFR inhibition blocks oncogenic signaling while Remodelin disrupts the post-transcriptional programs that support tumor cell survival and immune evasion.

In HCC, targeting NAT10 overcomes PARP inhibitor resistance by attenuating homologous recombination repair (169), suggesting that NAT10 inhibitors may be combined with PARP inhibitors to treat HCC and potentially other cancers with homologous recombination deficiency. This finding adds DNA repair regulation to the growing list of NAT10 functions in cancer and suggests that its inhibition may sensitize tumors to genotoxic therapies.

The ability of Remodelin to suppress HIF-1α expression (75) suggests potential synergy with anti-angiogenic agents such as bevacizumab or apatinib, as NAT10 inhibition may reduce the HIF-1α-driven angiogenic program that limits the efficacy of VEGF-targeted therapies.

### 9.4 Overcoming chemotherapy resistance

NAT10-mediated ac4C modification contributes to chemotherapy resistance in multiple cancer types through diverse mechanisms, and targeting NAT10 may overcome this resistance by disrupting the post-transcriptional programs that support drug tolerance.

The mechanisms by which ac4C modification drives chemotherapy resistance are diverse, but a consistent pattern emerges: NAT10 activity supports multiple resistance programs simultaneously, and its inhibition can reverse resistance across drug classes. Where resistance is driven by enhanced DNA repair, as in cisplatin-resistant bladder cancer, where NAT10 promotes ac4C-associated repair pathway activity, Remodelin restores drug sensitivity by impairing this repair mechanism (170). Where resistance is driven by EMT, as with doxorubicin in breast cancer and HCC, Remodelin reverses the mesenchymal phenotype and restores chemosensitivity (165). Where resistance involves drug efflux or metabolic adaptation, as in capecitabine-resistant breast cancer, NAT10 inhibition again restores susceptibility (171). This mechanistic versatility suggests that NAT10 functions as a broad enabler of chemotherapy resistance rather than a resistance mechanism specific to any single drug or pathway, and that its inhibition may be a generalizable chemosensitization strategy applicable across drug classes and tumor types.

### 9.5 Novel NAT10 inhibitors and delivery strategies

While Remodelin has shown promising preclinical efficacy, its pharmacokinetic properties may limit clinical translation, motivating efforts to develop novel NAT10 inhibitors and delivery strategies with improved drug-like properties. The synthesis of [11C]Remodelin as a positron emission tomography (PET) probe for imaging NAT10 expression *in vivo* (166) represents an important step toward clinical translation, as it may facilitate patient selection and treatment monitoring in future clinical trials. Additionally, the identification of Panobinostat, an FDA-approved HDAC inhibitor, as an indirect inhibitor of NAT10-mediated ac4C modification in HCC (172) suggests that existing approved drugs may be repurposed to target the ac4C pathway, potentially accelerating clinical translation.

A particularly pressing challenge for clinical translation is the cell-type-specific paradox of NAT10 inhibition: while suppressing NAT10 in tumor cells is therapeutically beneficial, systemic NAT10 inhibition may impair the anti-tumor function of myeloid cells, as demonstrated in HCC. To address this challenge, tumor-targeted delivery strategies are essential. Ligand-conjugated nanoparticles represent a promising approach: nanoparticles functionalized with tumor-targeting ligands such as folate, transferrin, or tumor-specific antibody fragments (e.g., anti-HER2, anti-EGFR) can selectively deliver NAT10 inhibitors to malignant cells while minimizing exposure in myeloid cells. Lipid nanoparticles (LNPs) encapsulating Remodelin or next-generation NAT10 inhibitors, decorated with tumor-homing peptides or antibody fragments, have shown proof-of-concept efficacy in preclinical models of targeted epitranscriptomic therapy. Antibody-drug conjugates (ADCs) represent another strategy: conjugating NAT10 inhibitors to antibodies targeting tumor-specific surface antigens (e.g., HER2, TROP2, or folate receptor α) would enable selective intracellular delivery of the inhibitor payload specifically to antigen-expressing tumor cells. Additionally, tumor microenvironment-responsive nanocarriers, designed to release their payload in response to the acidic pH, elevated reactive oxygen species, or matrix metalloproteinase activity characteristic of the TME, could further enhance tumor selectivity. These targeted delivery approaches would allow exploitation of the therapeutic benefits of NAT10 inhibition in tumor cells while preserving NAT10 function in myeloid cells and other immune cell populations, potentially converting a systemic liability into a tumor-selective therapeutic advantage (93, 173).

### 9.6 NAT10 as a prognostic biomarker

Beyond its role as a therapeutic target, NAT10 expression may serve as a prognostic biomarker across multiple cancer types, reflecting the broad importance of ac4C modification in cancer biology.

High NAT10 expression consistently correlates with poor prognosis, aggressive tumor phenotypes, and reduced immune cell infiltration across cancer types, a pattern that reflects the dual metabolic and immunological pro-tumorigenic functions described throughout this review (174). Importantly, the prognostic signal may be refined by examining the ac4C modification landscape rather than NAT10 expression alone: ac4C-modified gene signatures predict tumor prognosis and immune response in colon adenocarcinoma (175), suggesting that transcriptome-wide ac4C profiling could provide more nuanced prognostic information than a single protein biomarker. Realizing this biomarker potential will require the development of standardized, clinically validated assays for measuring NAT10 expression and ac4C modification levels in tumor samples, a prerequisite for patient stratification in future clinical trials.

### 9.7 Cancer-specific research progress

**Table 3** summarizes the current research progress on ac4C modification across different cancer types, highlighting the metabolic and immune mechanisms involved and the therapeutic strategies evaluated.

**Table 3:** Summary of ac4C modification research across cancer types.

Cancer type	Key targets	Metabolic effects	Immune effects	Therapeutic strategies
Gastric cancer	HK2, LDHA, SEPT9, HIF-1α	Enhanced glycolysis; HIF-1α stabilization	Not directly studied	Remodelin; anti-angiogenic
NSCLC	ENO1, KPNB1, GAS5	Glycolysis via ENO1	PD-L1 nuclear translocation; IFN-I	Remodelin; ICI combination
TNBC	JunB, LDHA, MDR1, BCRP	Glycolysis via JunB/LDHA	Immunosuppressive TME	Remodelin; doxorubicin
HCC	HSP90AA1, eEF2/HMGB2	ER stress effects	Cell type-specific PD-L1	Remodelin; lenvatinib; PARP-i
Colorectal cancer	PD-L1	Kynurenine metabolism	Kynurenine-AhR-NAT10-PD-L1	Remodelin + EGFR-i
Cervical cancer	FOXP1	Glycolysis via FOXP1	Immunosuppression via FOXP1	Remodelin + anti-PD-1
Ovarian cancer	PGAM1	Glycolysis; stemness	Not directly studied	NAT10/ac4C/PGAM1 targeting
NPC	DDX5, HMGB1	Not directly studied	T cell suppression	Remodelin + anti-PD-1
Pancreatic cancer	Multiple targets	Not directly studied	CD8+ T cell modulation	NAT10-i + anti-PD-1
Prostate cancer	DNA replication factors	Not directly studied	CD8+ T cell suppression	Remodelin
Osteosarcoma	LDHA, PFKM	ac4C-m6A crosstalk	Not directly studied	ac4C-m6A axis targeting
HNSCC	GLMP	MAPK/ERK activation	TME remodeling; MDSC	NAT10 as biomarker
Bladder cancer	DNA repair genes	Not directly studied	Not directly studied	Remodelin; cisplatin
Retinoblastoma	HK1	Glycolysis via HK1	Not directly studied	NAT10/ac4C/HK1 targeting
Colon adenocarcinoma	ac4C-modified genes	Not directly studied	Immune response; MSI	NAT10 as biomarker

NSCLC, non-small cell lung cancer; TNBC, triple-negative breast cancer; HCC, hepatocellular carcinoma; NPC, nasopharyngeal carcinoma; HNSCC, head and neck squamous cell carcinoma; ICI, immune checkpoint inhibitor; PARP-i, PARP inhibitor; EGFR-i, EGFR inhibitor; NAT10-i, NAT10 inhibitor; MSI, microsatellite instability.

## 10 Conclusion and future perspectives

### 10.1 Summary of key findings

This review has comprehensively examined the roles of N4-acetylcytidine (ac4C) modification in tumor metabolism and immunity, with a particular focus on the crosstalk between these two fundamental cancer hallmarks. The evidence reviewed here supports a model in which NAT10-mediated ac4C modification functions as a molecular bridge that simultaneously drives metabolic reprogramming and immune evasion, creating a self-reinforcing pro-tumorigenic state that is particularly resistant to single-modality therapeutic interventions.

Several key findings emerge from this synthesis. NAT10 is the only identified enzyme capable of catalyzing ac4C modification on mRNA, making it an attractive therapeutic target whose inhibition can simultaneously disrupt multiple downstream programs. The lack of identified readers and erasers distinguishes ac4C from the m6A modification system and raises fundamental questions about the reversibility and dynamic regulation of this modification that remain to be resolved.

NAT10-mediated ac4C modification enhances the stability and translation of key glycolytic enzymes, including HK1/2, ENO1, LDHA, and PGAM1, driving the Warburg effect across multiple cancer types. The NAT10/SEPT9/HIF-1α positive feedback loop further amplifies glycolytic reprogramming by sustaining HIF-1α activity, creating a self-reinforcing metabolic program that persists even under normoxic conditions. The conservation of these mechanisms across gastric, lung, breast, ovarian, and other cancer types suggests that ac4C-driven glycolytic reprogramming is a general feature of malignancy.

ac4C modification promotes immune evasion through multiple mechanisms, including upregulation of PD-L1 expression via at least three distinct pathways, suppression of T cell function through the DDX5/HMGB1 axis, inhibition of type I interferon signaling via the MYC/CDK2/DNMT1 pathway, and remodeling of the tumor microenvironment through GLMP/MAPK/ERK signaling. The breadth of these immune-evasive functions underscores the central importance of NAT10 in tumor immunology and suggests that its inhibition could achieve broader immunological effects than targeting any individual downstream pathway.

Critically, ac4C modification serves as a molecular bridge connecting metabolic reprogramming to immune escape through several interconnected axes: the NAT10/ac4C/FOXP1 axis couples glycolytic gene activation to immunosuppressive molecule suppression; the kynurenine-AhR-NAT10-PD-L1 axis links tryptophan catabolism to immune checkpoint upregulation; the NAT10/SEPT9/HIF-1α loop sustains both glycolytic enzyme expression and HIF-1α-driven PD-L1 upregulation; and glycolysis-derived lactate directly suppresses anti-tumor immunity while promoting Treg expansion. This metabolic-immune integration explains why tumors with high NAT10 activity are simultaneously metabolically hyperactive and immunologically suppressive.

The therapeutic potential of targeting NAT10 is supported by extensive preclinical evidence demonstrating that Remodelin and other NAT10 inhibitors reduce tumor growth, reverse EMT, overcome chemotherapy resistance, and synergize with immune checkpoint inhibitors across multiple cancer types. The dual metabolic-immune functions of NAT10 suggest that its inhibition may achieve synergistic anti-tumor effects that cannot be replicated by targeting either hallmark individually, positioning NAT10 inhibitors as uniquely valuable agents in combination therapy regimens.

### 10.2 Current challenges

Despite significant progress, several challenges remain in the field of ac4C biology that must be addressed before NAT10-targeted therapies can be translated to the clinic.

The most fundamental gap is the absence of identified ac4C readers and erasers. Unlike the well-characterized m6A modification system, no dedicated ac4C readers or erasers have been definitively identified, limiting our understanding of how ac4C modification is recognized and reversed. Resolving this question is essential for understanding the full regulatory logic of ac4C modification and for identifying additional therapeutic targets within the ac4C regulatory machinery.

Current ac4C detection methods also have important limitations. acRIP-seq lacks single-nucleotide resolution, RedaC:T-seq requires careful optimization to minimize background, and LC-MS/MS cannot identify specific modification sites. The development of more advanced detection technologies, including single-molecule sequencing approaches that enable direct, base-resolution detection of ac4C without chemical treatment, will be essential for comprehensively characterizing the ac4C landscape in cancer and for identifying novel ac4C targets.

The cell type-specific effects of ac4C modification represent another important challenge. As demonstrated in HCC, ac4C modification may have opposing effects in different cell types within the tumor microenvironment, promoting immune evasion in tumor cells while supporting anti-tumor immune function in myeloid cells. This complexity necessitates the development of cell type-targeted delivery strategies for NAT10 inhibitors to maximize therapeutic benefit while minimizing unintended effects on immune cell function.

Clinical translation of NAT10 inhibitors faces additional challenges related to pharmacokinetics and selectivity. Remodelin has shown promising preclinical efficacy, but its pharmacokinetic properties and potential off-target effects may limit its clinical utility. The development of more potent, selective, and drug-like NAT10 inhibitors with favorable pharmacokinetic profiles will be essential for clinical translation, and structure-based drug design enabled by the available crystal structure of NAT10 provides a rational path forward.

Finally, the development of standardized biomarker assays for measuring NAT10 expression and ac4C modification levels in clinical samples remains an unmet need. Although NAT10 expression correlates with prognosis in multiple cancer types, standardized assays for clinical use have not been developed or validated, limiting the ability to identify patients most likely to benefit from NAT10-targeted therapies and to monitor treatment response.

### 10.3 Future research directions

Based on the current state of knowledge and identified challenges, several priority research directions emerge for the field.

The identification of ac4C readers and erasers should be a top priority. Systematic proteomic screens using ac4C-modified RNA as bait, combined with genetic screens for modifiers of ac4C-dependent phenotypes, could identify proteins that specifically recognize or remove ac4C modification. Such discoveries would fundamentally advance our understanding of ac4C biology and reveal new therapeutic targets. Specifically, RNA pull-down experiments using biotinylated ac4C-modified RNA oligonucleotides as bait, followed by quantitative proteomics (e.g., SILAC or TMT-based mass spectrometry), would enable unbiased identification of proteins that selectively bind ac4C-containing sequences. Complementary CRISPR-Cas9 genome-wide loss-of-function screens, using ac4C-dependent phenotypes such as mRNA stability, translational output, or drug sensitivity as readouts, could identify candidate reader proteins whose depletion phenocopies ac4C loss. For eraser identification, biochemical fractionation of cellular extracts combined with ac4C deacetylase activity assays using synthetic ac4C-modified RNA substrates would provide a functional readout for eraser discovery. These systematic approaches, modeled on the successful identification of m6A readers (YTHDF1/2/3, IGF2BPs) and erasers (FTO, ALKBH5), provide a clear and actionable roadmap for completing the ac4C regulatory machinery and enabling the development of reader- or eraser-targeted therapeutic strategies (45).

The development of single-cell and spatially resolved ac4C detection methods will enable characterization of ac4C modification patterns in different cell types within the tumor microenvironment, addressing the critical question of how ac4C modification varies across the cellular heterogeneity of tumors. Such approaches will be essential for understanding the cell type-specific consequences of NAT10 inhibition and for designing targeted delivery strategies.

Further mechanistic studies are needed to fully elucidate how ac4C modification coordinates metabolic reprogramming and immune evasion, particularly to identify additional nodes of metabolic-immune crosstalk beyond the FOXP1, kynurenine, HIF-1α, and lactate axes described in this review. Systems-level approaches integrating transcriptomics, metabolomics, and immune profiling will be particularly valuable for mapping the full landscape of ac4C-mediated metabolic-immune integration.

Medicinal chemistry efforts should focus on developing more potent, selective, and drug-like NAT10 inhibitors. Structure-based drug design, enabled by the available crystal structure of NAT10, provides a rational path for optimizing inhibitor potency and selectivity. Targeted delivery strategies, including nanoparticle formulations and antibody-drug conjugates, should be explored to achieve tumor cell-selective NAT10 inhibition while sparing immune cells.

Standardized assays for measuring NAT10 expression and ac4C modification levels should be developed and validated in clinical cohorts to facilitate patient stratification and treatment monitoring. Prospective biomarker studies embedded within clinical trials will be essential for establishing the predictive value of NAT10 expression for response to NAT10-targeted therapies and for identifying resistance mechanisms.

Based on the strong preclinical rationale, clinical trials evaluating NAT10 inhibitors alone or in combination with immune checkpoint inhibitors should be initiated. Careful patient selection based on NAT10 expression, tumor metabolic phenotype, and immune microenvironment characteristics will be essential for maximizing the probability of clinical success. Basket trial designs that enroll patients across cancer types based on NAT10 expression rather than histology may be particularly appropriate given the pan-cancer relevance of NAT10-mediated ac4C modification.

While this review has focused on cancer, ac4C modification may also play important roles in other diseases, including autoimmune disorders, infectious diseases, and neurological conditions, where NAT10 expression and activity have been reported to be dysregulated. Exploring the role of ac4C modification in these contexts may reveal new therapeutic opportunities and deepen our understanding of the fundamental biology of this modification.

### 10.4 Concluding remarks

N4-acetylcytidine modification has emerged as a critical regulator of cancer biology, orchestrating both metabolic reprogramming and immune evasion. The identification of ac4C as a molecular bridge connecting these two cancer hallmarks provides new insights into tumor biology and opens new avenues for therapeutic intervention. Targeting NAT10-mediated ac4C modification, particularly in combination with immunotherapy, holds promise for improving cancer treatment outcomes. Continued research efforts to address current challenges and pursue the outlined future directions will be essential for realizing the full therapeutic potential of targeting ac4C modification in cancer.
